# Advanced oxidation processes for pesticide degradation: a comprehensive review on the role of nano zero-valent metals and persulfate activation

**DOI:** 10.1039/d5ra06043e

**Published:** 2025-10-27

**Authors:** Muhammad Aftab, Zia Ul Haq Khan, Noor Samad Shah, Fida Ullah, Syed Khasim

**Affiliations:** a Department, of Chemistry, COMSATS University Islamabad Park Road Islamabad 45550 Pakistan Zia.khan@comsats.edu.pk ziaulhaqkhan11@gmail.com; b Department of Chemistry, COMSATS University Islamabad Abbottabad Campus KPK Pakistan; c Advanced Materials Research Laboratory, Department of Physics, Faculty of Science, University of Tabuk Tabuk 71491 Saudi Arabia

## Abstract

The widespread use and persistence of pesticides in aquatic environments pose a severe risk to ecosystems and human health. This review comprehensively analyses advanced oxidation processes (AOPs) for pesticide degradation, focusing on and persulfate activation mediated by nano zero-valent metals (nZVMs). Recent studies highlight the exceptional performance of nano zero-valent iron (nZVI), zinc (nZVZn), and copper (nZVCu) in generating reactive oxygen species (ROS) such as hydroxyl and sulfate radicals that effectively degrade persistent organic pollutants, including chlorpyrifos, atrazine, and *p*-chlorophenol. The paper further examines the mechanisms underlying pollutant degradation, the effects of operational parameters such as pH, oxidant and catalyst dosage, and the synergistic role of composite systems like nZVI/BC and nZVZn/PMS. In addition, degradation pathways and mineralization efficiencies are discussed in detail, providing insight into the reaction kinetics and mechanistic transformations of target pollutants. This review not only summarizes the advantages of integrating persulfate-based AOPs with nZVM catalysts but also identifies key challenges such as catalyst recovery, secondary pollution, and scalability. Overall, the findings provide a framework for advancing sustainable, efficient, and eco-friendly AOP-based technologies for pesticide remediation.

## Abstract 2

The widespread use and persistence of pesticides in aquatic environments pose a severe risk to ecosystems and human health.

## Graphical abstract

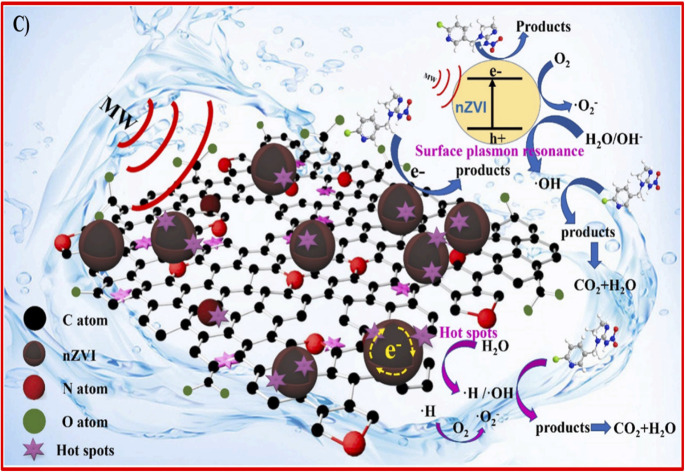

## 1. Introduction

**Fig. 1:**
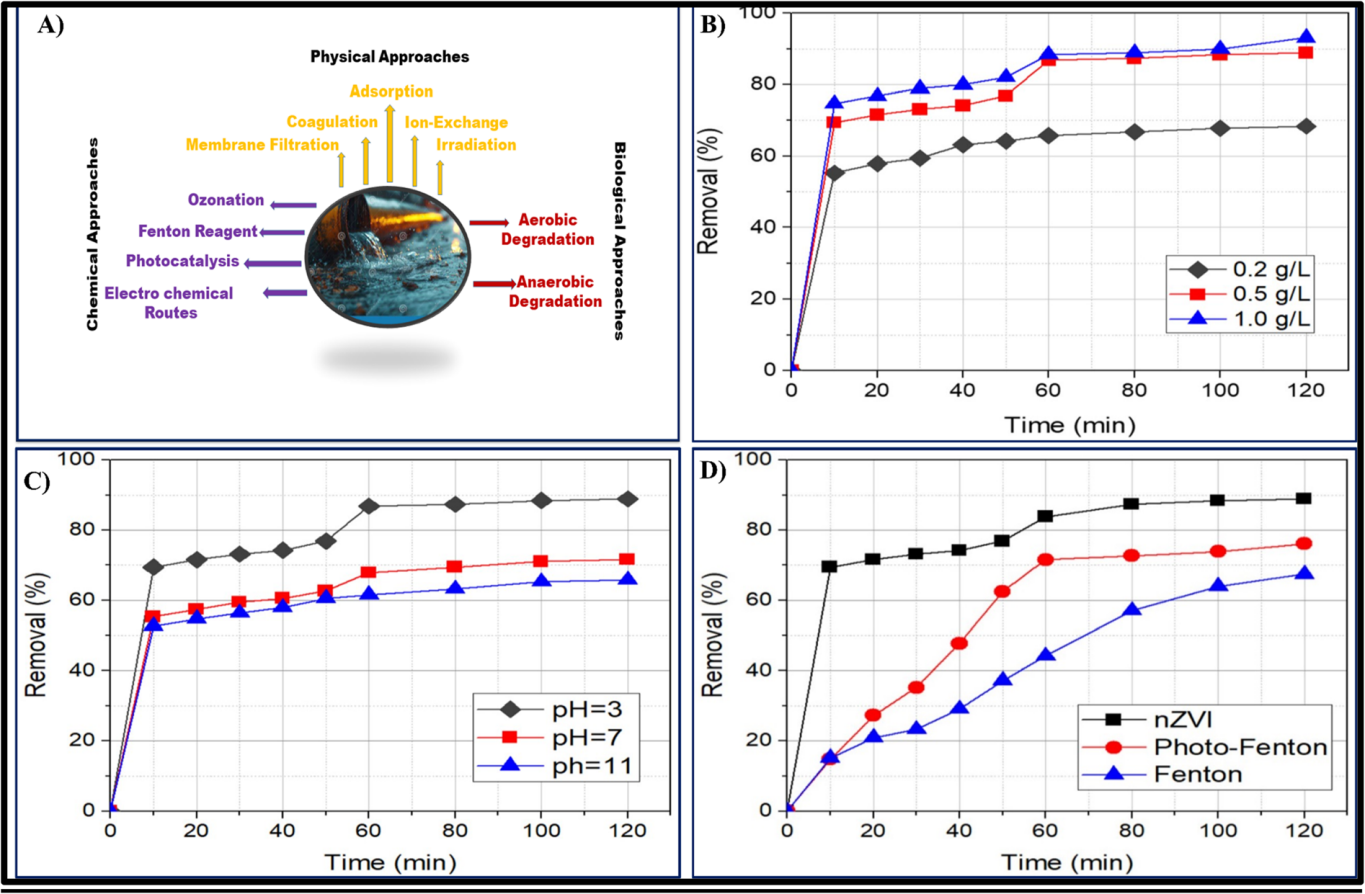
(A) Chemical, biological, and physical approaches to remove or degrade the organic pollutants in the wastewater. (B) Effect of nZVI dosage on the degradation of 2,4-D.^1^ (C) Effect of pH on the degradation of 2,4-D.^1^ (D) Comparison of degradation processes for the removal of 2,4-D.^1^

The increasing use and persistence of pesticides in the environment have raised significant concerns regarding their impact on ecosystems and human health. Organic pollutants, particularly pesticides, are highly resistant to degradation due to their complex chemical structures, making them difficult to remove from wastewater and environmental matrices.^1^ As a result, finding efficient and sustainable methods to degrade these pollutants is crucial for environmental protection, as some methods are illustrated in Fig. 1. Advanced oxidation processes (AOPs) have emerged as a promising approach for the removal of such organic contaminants. AOPs are based on the generation of highly reactive species, such as hydroxyl radicals (˙OH), sulfate radicals (˙SO_4_^−^), and other reactive oxygen species (ROS), which are capable of breaking down even the most persistent organic compounds.^2,3^ Among the various AOPs, Fenton, and persulfate activation systems have shown remarkable potential in degrading persistent organic pollutants.^4,5^ Recent advancements have emphasized the integration of nano zero-valent metals (nZVMs)—especially nano zero-valent iron (nZVI), zinc (nZVZn), and copper (nZVCu)—into AOP frameworks to enhance catalytic performance. These materials act as efficient electron donors and activators for oxidants such as hydrogen peroxide and persulfate, accelerating the generation of reactive radicals. In particular, nZVI has received extensive attention for its high surface area, redox activity, and ability to activate persulfate for pollutant degradation. Similarly, nZVZn and nZVCu demonstrate strong catalytic and reductive properties, making them effective in the degradation of pesticides such as chlorpyrifos, atrazine, and *p*-chlorophenol.^5–7^ These metals can be used effectively in combination with other processes, such as Fenton and persulfate activation, for enhanced degradation of pesticides.

Moreover, the incorporation of SI materials such as biochar (BC) further improves catalyst stability and dispersion, leading to enhanced oxidation efficiency and reduced aggregation. The synergistic interaction between nZVMs and persulfate systems has demonstrated superior degradation kinetics and mineralization rates, establishing these hybrid systems as leading candidates for large-scale wastewater remediation.^8–10^

This review systematically discusses the degradation of major pesticide groups using nano zero-valent metal-based AOPs, particularly focusing on persulfate activation. Key degradation mechanisms, operational parameters, and pathway analyses are presented, alongside critical evaluations of system efficiencies, challenges, and future perspectives. The aim is to consolidate current knowledge and provide a roadmap for developing sustainable and efficient AOP technologies for environmental decontamination.

## 2. Introduction to pesticides and their degradation pathway

Pesticides are organic contaminants found in water bodies. Pesticides enter the aquatic system primarily from three sources: (a) the farming industry, where they are used for insect management to protect harvests from pest destruction; (b) the effluents of industries that manufacture pesticides; and (c) home use. Typical examples of pesticides are insecticides, herbicides, fungicides, rodenticides, and cultivation regulators. Excessive use of pesticides endangering aquatic life as well as human wellness. With the exception of aldrin, heptachlor, dieldrin, and heptachlor epoxide, which are permitted within a range of 0.3 ppm, the World Health Organization states that the maximum allowable limit for pesticides in drinking water is 0.1 ppm of each pesticide and 0.5 ppm of total pesticides.^11,12^ Organic pesticides are artificial compounds, or mixtures of chemicals, intended to avoid, oversight, or eradicate any undesirable creatures, including fungus and pests of animals.^13^ In addition to being categorized into chemical families such as carbamates, sulfonylureas, triazines, chloroacetanilides, and the organochlorine.^14,15^ Researchers also classify pesticides depending on how they affect target organisms, such as growth promoters, nitrogen metabolism inhibitors, lipid generation inhibitors, and amino acid inhibitors. Because of their distinct mechanism for working and effective insecticidal properties, neonicotinoid insecticides are among the many types of insecticides that are gaining popularity swiftly.^16^ Regarding the safety of water, tetrachloroethylene (TMX), a subsequent insect killer, is considered to be one of the most harmful neonicotinoid pesticides.^3,17^ By using peroxides to create large numbers of effective chemicals, like sulphate and OH radicals, throughout the activation phase, advanced oxidation technologies (AOPs) can remove pollutants from the environment because of their significant oxidation capability. Transition metals, activated carbon, and ultrasonication are a few of the techniques used for activating persulfate (PS).^18^ Using extreme temperatures pyrolysis, biomass is converted into cheap carbonaceous material known as biochar (BC). Due to the abundance of surface functional groups in biochar, activated PS can more easily degrade organic pollutants. Nevertheless, every single biochar's activating efficiency is specifically weak.^3,19^ One valentine iron and manganese are common in nature, they are effective at adsorbing and catalysing a variety of contaminants due to their many sites of action, rapid transfer of electrons capabilities, and participation in several chemical processes. Since Fenton-mediated-bimetallic catalysts have richer valence states than oxides comprising monometallic elements, they offer superior PS activating characteristics.^20^ Applied at the nanoscale, nanotechnology is a rapidly developing sophisticated technology. Its primary focus is on using, managing, and comprehending the unique characteristics of material that can manifest at tiny scales, that span between 1 to 100 nm. There are multiple phases in nanocomposite substances including one, two, or three dimensions in nanometre scale.^21^ The distinctive qualities of nanocomposites cannot be achieved by any one of their individual components alone. The matrix is that part of a composite that contains vital quantity of constituents. The method of incorporating components to the matrix material to improve the attributes of nanocomposites is known as reinforcement. Reinforcement is a procedure that improves the chemical and physical characteristics of nanocomposites, which are typically composed of dissimilar components divided by a junction. The creation of nanocomposites determines how they're categorized. A variety of matrix substances and nanoparticles are employed in the manufacture of nanocomposites. Depending on the substrate or matrix substance utilized, there are three different kinds of nanocomposites.^22^ These are Metal Matrix Nanocomposites (MMNC), Polymer Matrix Nanocomposites (PMNC), and Ceramic Matrix Nanocomposites (CNMC). Because of their substantial surface to volume ratio, space among fillers, preferable structural capabilities, outstanding ductility without losing durability, scratch resistance, and altered optical characteristics, nanocomposites are preferable to traditional composite substances. A subclass of composite materials known as ceramic matrix nanoparticles (CMNCs) is composed of ceramic matrix reinforced with metal or ceramic fibers. CMNCs are designed to solve issues with traditional technological ceramics including silicon nitride, Al_2_O_3_, and Zr_2_O_3_*etc.*^23^ In the past ten years, a great deal of research has been done on nanoscale zero-valent iron (nZVI), which has demonstrated great promise for the treatment of organic pollutants in sewage.^24^ nZVI has special reactivity interfaces involving deposition and conversion of pollutants through reduction or oxidation mechanisms due to the core–shell arrangement of the Fe^0^ centre and iron oxide coating.^20^ nZVI is being utilized to eliminate a range of naturally occurring contaminants, such as halogen-containing organic substances. The inorganic contaminants the heavy metals ion and radioactive particles *e.g.*, U(vi).^25,26^ As a result, nZVI has emerged as the substrate of choice for natural product rehabilitation due to its abilities, simplicity, affordability, and ecological friendliness. NZVI nanoparticles nevertheless have drawbacks regardless of the benefits listed above, such as quick aggregation due to high magnetic attraction forces between particles.^27^ Furthermore, nZVI nanoparticles produce a protective barrier on their outermost layer when they readily interact with oxygen in solution and/or moisture.^28^ The agglutination and polishing features of nZVI are addressed by a variety of techniques, including: (1) encapsulating nZVI. bentonite and activated carbon; (2) covering the nZVI fragments with different minerals, and polymer compounds such as xanthan gum, and CTAB (cetyltrimethylammonium ammonium bromide);^29^ (3) injecting nZVI alongside an inert metal.^30^ The removal of naturally occurring contaminants by nZVI along with nanocomposite has been the subject of numerous studies lately the majority of which are application specific. For instance, Stefaniuk *et al.* (2016) primarily discussed the advantages and disadvantages of using nZVI, from its manufacture through its ecological uses.^31^ The effectiveness of nano-zero-valent nanoparticles (nZVP) made of various metals in degrading a broad variety of contaminants has been demonstrated.^32^ It is true that these particulates undergo oxidation, which leads to the reduction of contaminants (like nitrate);^33^ Cr VI;^33^ or oxygen, resulting in the production of hydrogen peroxide and the reactive cation *via* the original NZVP.^33^ Organic contaminants are swiftly and non-specifically attacked by OH radicals produced when H_2_O_2_ and a transition metal cation combine to initiate a Fenton-like reaction.^34^ With regard to environmental memory, nanosized zero valent iron (nZVI), in specific, have decided to use as a productive catalyst due to its intense activity.^35^ But because of their powerful magnetism and van der Waals interactions, nZVI nanoparticles not only have a tendency to clump together into larger-size fragments but also readily undergo oxidation, especially in anoxic conditions.^36,37^ nZVI has been deposited or adsorbed on a support (supporter) to address these drawbacks. This carrier can increase nZVI's surface area and dispersal, as well as partially ease oxidation and enhance nZVI's effectiveness regardless of usage or preservation.^38^ Because of their significant adsorption capacity towards contaminants, low cost, ease of preparation, and exceptional chemical and thermal resistance, carbon-based substances are perfect substrates for loading nZVI particles.^39,40^

### 2.1. Pesticides degradation by nZVMs

Some of the nZVMs and degradation methods are discussed as follows:

#### 2.1.1. nZVI-PF for degradation of 2,4 dichlorophenoxyacetic acid

2,4-Dichlorophenoxyacetic acid (2,4-D) is a pesticide that is widely employed in gardening and agricultural practices among the many agrochemicals now in use because of its inexpensive cost. Both its water solubility and biodegradability are relatively high. Due to its poor coefficient of soil adsorption, 2,4-D free acid can seep through the soil and perhaps leak into groundwater. In drinking water, a maximum allowable concentration of 100 parts per billion is permitted, with the World Health Organization (WHO) classifying it as moderately hazardous.^41^ Aromatic compounds, colors, medicines, detergents, herbicides, insecticides, and other dissolved organic contaminants can be removed from contaminated waters using advanced oxidation processes (AOPs).^42,43^ Strong oxidants are produced quickly by the reaction of oxygen and nanoscale zero-valent iron (nZVI). First, two electrons are transferred from Fe^0^ surfaces to O_2_, oxidizing ferrous iron (Fe^2+^) and producing H_2_O_2_ (eqn (1)). Further two-electron exchange from ZVI can decrease the H_2_O_2_ to water molecules (eqn (2)). Hydroxyl radicals (˙OH), which have a strong oxidizing power against many organic molecules, are produced when Fe^2+^ is oxidized in the Fenton reaction (eqn (3)). In the photo-Fenton process, wherein Fe^3+^ is reduced to Fe^2+^, the photo OH radical is mostly formed *via* (eqn (4)). OH radicals are produced when UV light is irradiated because of Fe^3+^ catalysis.^44^

1 Fe^0^ + O_2_ + 2H^+^ → Fe^2+^ + H_2_O_2_

2 Fe^0^ + H_2_O_2_ + 2H^+^ → Fe^2+^ + 2H_2_O

3 Fe^2+^ + H_2_O_2_ → Fe^3+^ + ˙OH + OH^−^

4 Fe^3+^ + H_2_O + *hv* → Fe^2+^ + H^+^ + ˙OH

Fenton reaction's main issues include rapid H_2_O_2_ consumption, incomplete pollution mineralization, Fe ion loss, a narrow pH range, and low chemical degradation ratios.^45^ Alternative approaches have been presented for resolving similar issues.

There have been reports of use nZVI recently to clean up environmental contaminants. Due to their enormous surface areas and high surface reactivity, ZVI particles offer a cost-effective solution for even the most difficult environmental remediation issues. Nitrate, heavy metals, nitroaromatics, arsenic, dyes, phenol, and chlorinated organic compounds are just a few of the environmental pollutants that can be detoxified and transformed utilizing nZVI particles, according to published research.^46^ Simultaneously, supported and catalyzed modified nanoscale iron particles were employed to improve the remediation efficiency and reaction time. The BET-N_2_ technique yielded a specific surface area value of 44.7 ± 0.4 m^2^ g^−1^ for the nZVI. According to reports in the literature, BET surface areas were discovered to be 25 m^2^ g^−1^, 29.67 m^2^ g^−1^, 10.5 m^2^ g^−1^, and 36.5 m^2^ g^−1^.^47^ On the other hand, compared to nanoscale iron reported in the literature, the specific surface area of commercial Fe powder (<10 μ) is 0.9 m^2^ g^−1^.^47^ The cleanup process is currently underway at the iron surface. Several factors that affect degradation of 2,4-D by nZVI are listed below:

##### 2.1.1.1. nZVI dosage affect

Different concentrations of nZVI (0.2 g L^−1^, 0.5 g L^−1^, and 1.0 g L^−1^) were used to assess the impact of nZVI dosage on the 2,4-D remediation rate. Fig. 1(B) presents the results that were obtained. The effectiveness of 2,4-D remediation rose significantly with the addition of more and more nZVI particles. However, considerable removal was not seen at a dosage of 0.5 g L^−1^ nZVI, and this dosage is recommended as the ideal dosage because it is the most economical. As an electron donor, nZVI is crucial for initiating the breakdown of 2,4-D. More sources of Fe(ii) ions are present with higher dosages of nZVI. High nZVI densities also enable more active regions where the reaction takes place. Consequently, the reaction's efficiency is raised.

##### 2.1.1.2. pH effect

One important factor in the Fenton technique is the initial pH impact. A pH range of 3 to 11 was used for the investigation. The pH of the solution served as the sole variable in experiments conducted under the same conditions. Lowering the pH value was found to enhance the rate of deterioration. However, the degradation efficiency tends to decline at pH = 3, because the nZVI surface becomes protonated. The ideal pH range for excellent elimination effectiveness is 3–5, according to earlier research.^48^ This evidence leads to the conclusion that pH = 3 is the ideal pH for the degradation of 2,4-D as shown in Fig. 1(C).

##### 2.1.1.3. Degradation method selection

The removal effectiveness of the Fenton, photo-Fenton, and nZVI procedures was compared as shown in Fig. 1(D). Fe^2+^ to H_2_O_2_ ratio of 1 : 5 was chosen. The ideal ratio for Fenton and photo-Fenton studies was found to be this one.^49^ Compared to the other two techniques, the degradation of 2,4-D using nZVI has shown more effective. This is a result of nZVI's strong catalytic activity in breaking down 2,4-D. Fenton and photo-Fenton remained at 67% and 76%, respectively, after 89% of them were removed using the nZVI technique. The following sequence of techniques was found to be the most efficient in eliminating 2,4-D: ZVI > photo-Fenton > Fenton.

The purpose of the comparison is to determine how well nanoscale zero-valent iron (nZVI) removes the chlorinated herbicide 2,4-D from polluted soil and water. The initial pH of the solution and the nZVI dosage are the primary factors influencing the elimination rate. The comparison shows that while elimination efficiency is improved by increasing nZVI dosage, this effect plateaus at a particular concentration. The best dose for treating patients economically is determined to be 0.5 g L^−1^. Furthermore, there is a considerable increase in the degradation rate when the pH is lowered from 11 to 3. According to the study, nZVI is a more effective option for 2,4-D remediation than both photo- and classical Fenton procedures.

#### 2.1.2. nZVI-PF for degradation of pirimicarb

As one of the most widely used pesticide groups in the world, carbamates continue to pose a hazard to the environment because of their many effective biological activities as fungicides, insecticides, and molluscicides.^50^ The primary issue with carbamates is how long they take to disappear from the environment some of the compounds might linger for years after they are used. These substances also have a high solubility in water, which means that their leftovers will inevitably find their way into ground and surface waters through leaching and runoff from soil. It is discovered that these residues are becoming more prevalent in environmental matrices concurrently with the extensive use of carbamates in agriculture. Carbamates are known to have toxicological effects on humans in addition to their impacts on the environment because they inhibit acetylcholinesterase.^51^ More specifically, one of the carbamate derivatives that is extensively used as an insecticide for aphids in fruits and vegetables is 2-dimethylamino-5,6-dimethylpyrimidin-4-yldimethylcarbamate, or pirimicarb. Just like other carbamate compounds, pirimicarb inhibits acetylcholinesterase, making it extremely hazardous to mammals. Pirimicarb is a possible mutagen and carcinogen, according to several research. Soloneski and Larramendy,^52^ for instance, used Chinese hamster ovary (CHO-K1) cells to show the genotoxicity and cytotoxicity of this pesticide. Class II (moderately harmful) is how the World Health Organization (WHO) has classified pirimicarb. A significant amount of study is required to find effective and straightforward methods for removing pirimicarb, particularly since this dangerous substance is frequently found in environmental waters due to its widespread use.^53^ A number of oxidation and reduction reactions have led to increased interest in nano-zero valent particles (nZV), particularly iron (nZVI), as a simple and non-selective agent for the elimination of organic materials. These responses lead to the designation of nZVI used asAOPs. nZVI have, in fact, shown activity against the self-generation of hydroxyl radicals (OH), which target organic materials aggressively and rapidly. This is due to nZVI's natural behavior in an oxygenated environment described in (eqn (5)), where it produces the Fenton reagents and, as a result, the Fenton reaction takes place as shown in (eqn (6)). As a result, nZVI serves as a steady source of the Fenton processes.^54^ A portion of the self-generated H_2_O_2_ may react with Fe^0^ (nZVI) as described in (eqn (7)), which would reduce H_2_O_2_ while serving as a source of Fe^2+^. Because of this, some writers have looked into the possibility of including more H_2_O_2_.^55^

5 Fe^0^ + O_2_ + 2H^+^ → Fe^2+^ + H_2_O_2_

6 Fe^2+^ + H_2_O_2_ → Fe^3+^ + HO˙ + HO^−^

7 Fe^0^ + H_2_O_2_2H^+^ → Fe^2+^ + H_2_O

Furthermore, direct photolysis of the target pollutant or its byproducts, homolytic breakage of H_2_O_2_ (achieved by UVC radiation), and regeneration of Fe^2+^ from spent Fe^3+^ (produced during the Fenton process eqn (6), which results in both ˙OH generation and Fe^2+^ constant supply (photo-Fenton process). (eqn (8)). are other ways that the addition of radiation can improve the process performance.

8 Fe^3+^ + H_2_O + *hv* → Fe^2+^ + H^+^ + HO˙

nZVI's is suggested as an affordable, expedient, and eco-friendly fix. Thus, agro-industrial leftovers are valued more while also making the synthesis of nZVI more feasible. In keeping with the tenets of the circular economy, this keeps these wastes from building up over huge tracts of land.

##### 2.1.2.1. Degradation mechanism of pirimicarb

**Fig. 2:**
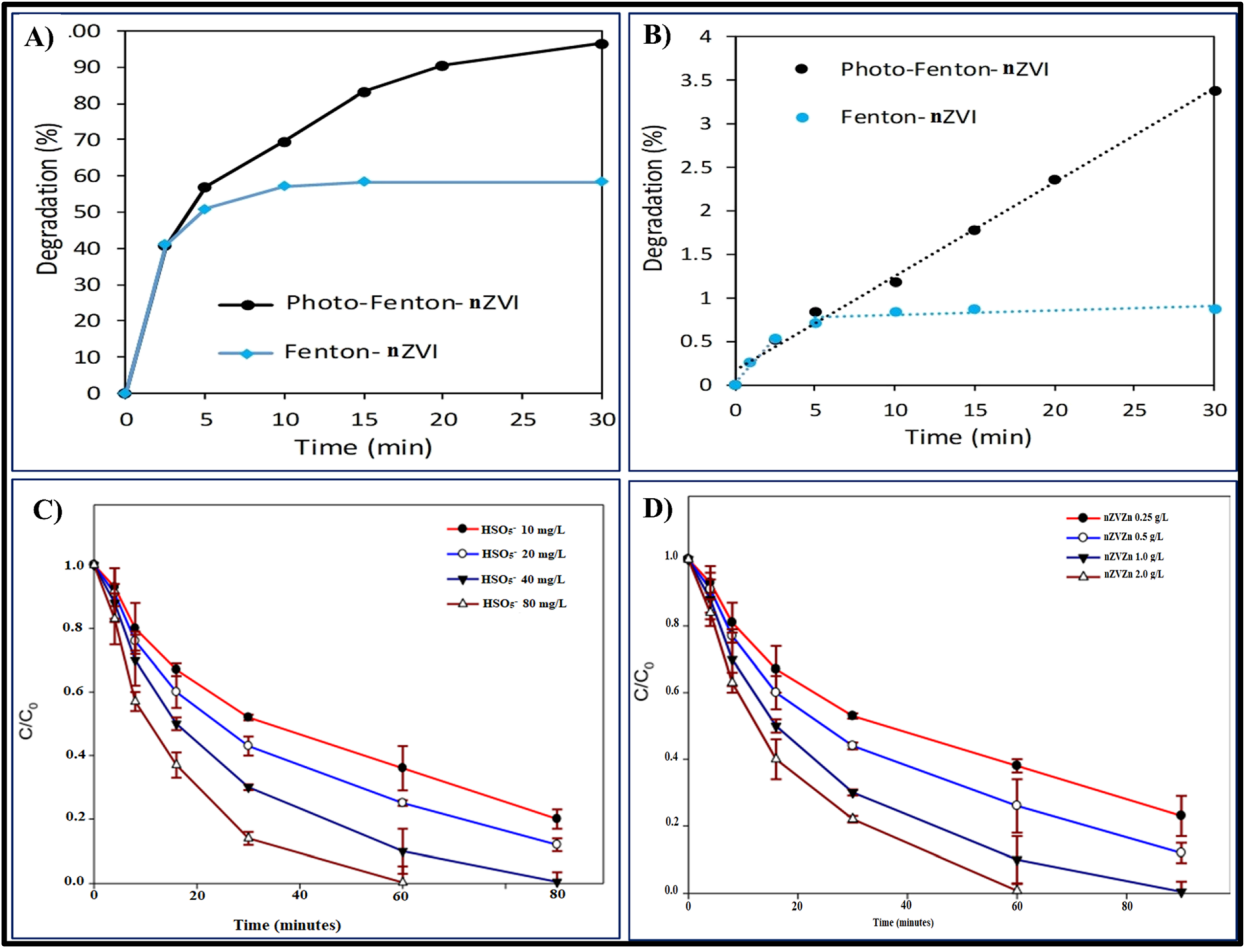
(A) Comparison on pirimicarb degradation by the Fenton-like process with nZVI 0.16 mM and 0.08 mM H_2_O_2_ and when being under darkness conditions or solar radiation. (B) Removal of CPY using HSO_5_^−^ catalyzed by Zn^0^ at varying HSO_5_^−^ doses. Conditions for the experiment: pH is 2.5, flow rate is 0.2 L min^−1^, [CP]_0_ = 10.0 mg L^−1^, [HSO_5_^−^] = 10–80 mg L^−1^, and [Zn^0^]_0_ = 1.0 g L^−1^.^2^ (C) Removal of CP using HSO_5_^−^ catalyzed by Zn^0^ at varying Zn^0^ concentrations. Conditions for the experiment: pH = 2.5, flow rate = 0.2 L min^−1^, [CPY]_0_ = 10.0 mg L^−1^, [HSO_5_^−^]_0_ = 40 mg L^−1^, and [Zn^0^]_0_ = 0.25–2.0 g L^−1^.^2^ (D) Impact of starting CP concentrations on CP elimination by Zn^0^-catalyzed HSO_5_^−^. Conditions of the experiment: [CP]_0_ = 2.5–20 mg L^−1^, [HSO_5_^−^]_0_ = 40 mg L^−1^, [Zn^0^]_0_ = 1.0 g L^−1^, pH = 2.5, flow rate = 0.2 L min^−1^.^2^

The primary species responsible for pirimicarb degradation are ˙OH. The absence of these species on the bulb solution results in a 95% reduction in health hazards. Assuming that it is a photo-Fenton-like process in which ˙OH are successfully created and nZVI promotes its performance, this was expected. AOPs are also significantly impacted by superoxide radicals (O_2_˙/HO_2_˙).^55^ Removing these oxidants off the system resulted in a 67% reduction in deterioration in this instance as shown in Fig. 2(A and B). This is associated with the Fenton process, in which the accessible H_2_O_2_ and the generated Fe^3+^ lead to the production of O_2_, as represented in eqn (9) and (10). Additionally, nZVI has the ability to stimulate the production of superoxide radicals.^56^

9 Fe^3+^ + H_2_O_2_ → Fe^2+^ + HO_2_˙ + H^+^

10 HO˙ + H_2_O_2_ → HO_2_˙ + H_2_O

The breakdown of pirimicarb is also influenced by singlet oxygen, indicating that non-radical pathways can also play a part in the degradation of pesticides. Catalytic activation of H_2_O_2_ can produce these species. In conclusion, nZVI exhibited a modest photocatalytic behavior, which has been documented earlier.^57,58^

This conclusion can be drawn from the observation that 15% of the pirimicarb degradation was negatively impacted by EDTA-trapped holes. It has been established that ˙OH are the primary degradation species and that nZVI can produce ˙OH, which aids in the degradation of pesticides. The formation of ˙OH is nearly instantaneous due to the rapid AOPs. After that, its produced quantity is significantly decreased and maintained continuously. The cyclic regeneration generated by the photo-Fenton-nZVI process with nZVI is responsible for this oxidant stability, as has been previously shown on the rise in elimination of pesticides.^59^

##### 2.1.2.2. Degradation pathway

**Scheme 1:**
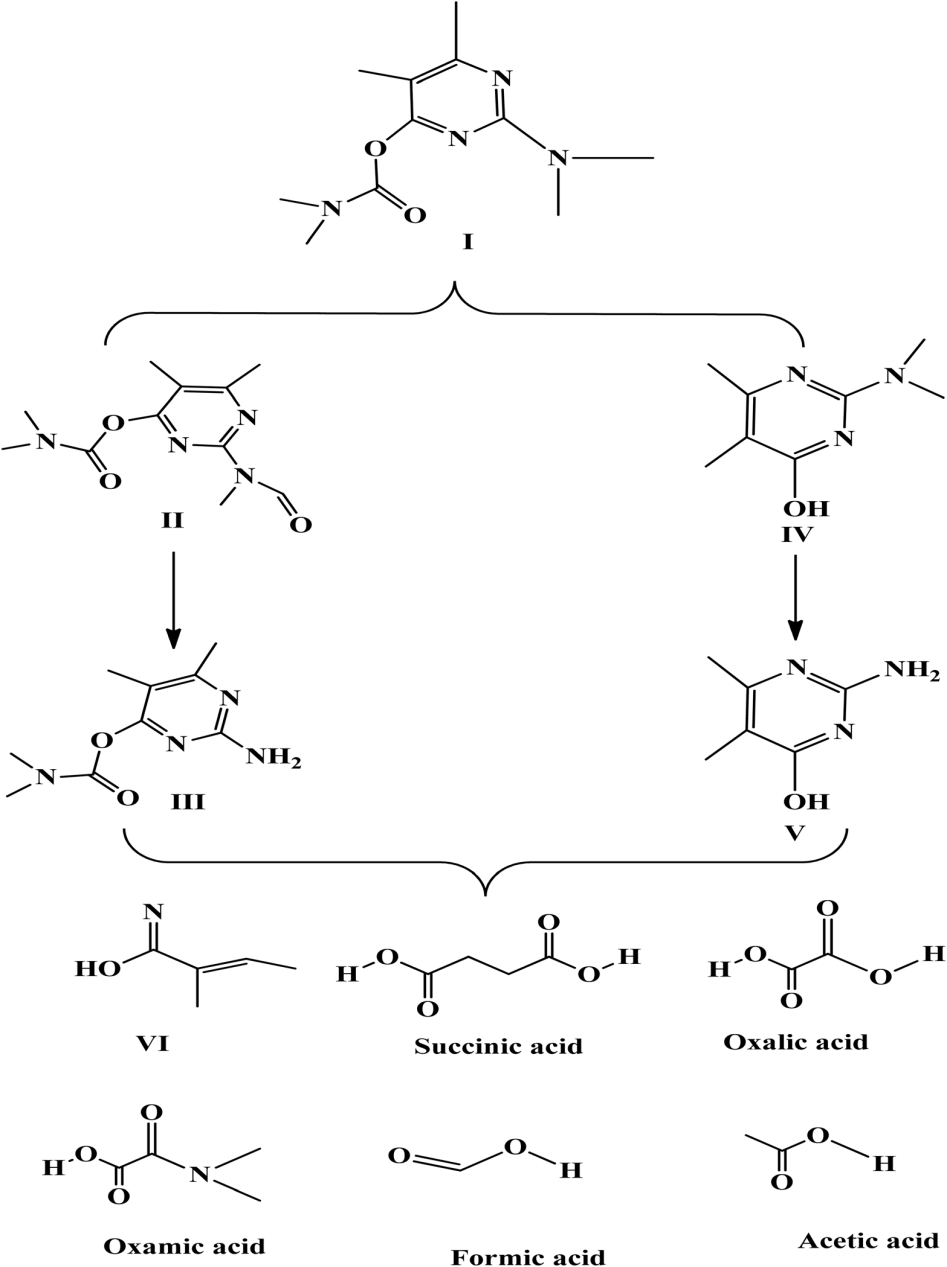
Proposed pirimicarb degradation under photo-Fenton-nZVI process.

The degradation by-products were measured (Table S1) and the suggested degradation pathway in order to confirm the degradation of pirimicarb and comprehend the mechanisms of degradation. Utilizing ˙OH on nucleophilic atoms (*N*-dimethyl group) is the basis for modifying pirimicarb to produce compound II.^60^ The methyl and carbonyl groups can be eliminated more easily and compound III can be reached by the introduction of a carbonyl bond, which may weaken the surrounding bonds. By breaking the dimethyl carbamate group on the pirimicarb molecule, chemical IV can be obtained.^61^ Because of the surrounding O groups' strong electronegativity, which weakens the aldehyde bond, this rupture occurs on that bond. *N*-dealkylation of compound IV is possible (compound V). Following that, compound VI is created by further breaks and potentially radical recombination Scheme 1.

#### 2.1.3. nZVZnC-PMS for degradation of chlorpyrifos

Nano-zerovalent zinc (nZVZn) is frequently used for the degradation of pesticides especially for chlorpyrifos due to several features; high reactivity and surface area, high reductive dechlorination, ecofriendly nature *etc.*^62^ Chlorpyrifos is a chlorine containing organophosphate pesticide that is commonly employed for killing and eradicating insects, parasites, and pests on a variety of crops, fruits and vegetables.^63^ It has been reported that the respiratory, cardiac, neurological, reproductive, and immune systems are all impacted by CPY contaminant in aquatic environments.^63,64^ Furthermore, DPs are produced when CPY degrades in an aquatic environment, and a few of these DPs are said to be extremely harmful. The incorporation of the reactive radicals, such as hydroxyl radical OH˙ and sulphate radical SO_4_˙^−^ in advanced oxidation technologies (AOTs) is very beneficial for the removal of harmful contaminants.^63,65^ Nevertheless, the water quality variables of the OH˙ containing AOTs (*i.e.*.HR-AOTs) eventually decrease their possible uses for dealing with persistent hazardous substances.^66^ Conversely, it is thought that the SO_4_˙^−^ mediated AOTs, or SR-AOTs, are not as affected by water quality standards and are also competent to remove a variety of stubborn toxic organic pollutants.^67^ In advance studies, persulfate anion such as peroxymonosulphate (HSO_5_˙^−^) that simultaneously produces hydroxyl radicals and persulphate radicals under metals undergoing heterolytic disintegration, irradiation, or the selected oxidizer is heating.^68^ Transition metals along with other activating processes are used less frequently to maximize the generation of hydroxyl and sulphate radicals from HSO_5_^−^ stimulation due to the consistent metal supply and elevated costs. On the other hand HSO_5_˙^−^ is said to be highly effectively activated into OH˙ and SO_4_˙^−^ by recently developed nano zerovalent metals (nZVMs, Ms^0^) technologies.^69^ For the activation of HSO_5_^−^ and other oxidants, nZVMs are preferred due to their inexpensiveness, ease of quenching reactive oxygen species (ROS), and progressive availability of metal ions.^69^ One of the most widely used nZVMs is nano zerovalent iron (nZVI), which is used to degrade harmful pollutants and activate HSO_5_˙^−^ and other oxidants. For the energizing of HSO_5_˙^−^ into hydroxyl radicals and persulphate radicals for the degradation of CPY, nano zerovalent zinc metal (nZVZn, Zn^0^) is chosen because of its inexpensiveness, environmentally friendly nature, and higher reduction potential than nZVI. CPY and total chlorine percent removals for each metal tested after 30 days of reaction, [CPY]_0_ = (103.0 ± 4.5) mg L^−1^, initial metal concentration = 0.5% (w/v), pH_0_ = 6.0 ± 0.2. Table S2.^70^

##### 2.1.3.1. Degradation pathway of CPY using Zn^0^ activated HSO_5_^−^

Chlorpyrifos breakdown is carried out under different conditions. To examine the roles of Zn^0^ and HSO_5_˙^−^ in chlorpyrifos breakdown, the following scenarios were considered: Zn^0^ alone, HSO_5_^−^ solely, Zn^2+^/HSO_5_˙, and zero-valent zinc with HSO_5_˙. At a fixed reaction period of 90 minutes, the elimination of CPY reached 10, 55, 78, and 99.5% for Zn^0^ alone, Zn^2+^ with HSO_5_^−^, and Zn^0^ with HSO_5_^−^ (Zn^0^/HSO_5_˙).^71^ When Zn^0^(s) meets dissolved oxygen, it oxidizes and becomes Zn_(aq)_^2+^ and aqueous electron (e_aq_^−^) as shown in eqn (11) and (12). When halogenated organic compounds (R–X) are involved, the aqueous electron serves as a reductant, such as chlorpyrifos causing their dehalogenation. The addition of NO_3_^−^ caused the degradation of CPY by Zn^0^ to decrease from 55% to 20%, which may have been caused by the high reactivity of NO_3_^−^ along with e_aq_^−^ and its competition with CPY for e_aq_^−^ as shown in eqn (13).

11 Zn^0^ → Zn^2+^ + 2e_aq_^−^

12 2e_aq_^−^ + R–X → X^−^ + R

13 NO_3_^−^ + 2e_aq_^−^ → NO_3_^2−^(*K*_s_ = 9.8 × 10^9^ M^−1^ s^−2^)

HSO_5_^−^ is catalysed by the metals Zn^2+^ and Zn^0^ in Zn^2+^ with HSO_5_^−^ and Zn^0^ with HSO_5_^−^ to produce OH˙ and SO_4_˙ as shown in eqn (14) and (15).^67,72^ Because of their potent oxidants and high oxidation potential, the COH and SO_4_˙ can easily attack target contaminants and convert them into DPs, as demonstrated by eqn (17).^73^

14 e_aq_^−^ + HSO_5_^−^ → SO_4_^2−^ + ˙OH

15 e_aq_^−^ + HSO_5_^−^ → ^−^OH + SO_4_˙^−^

16 ˙OH/SO_4_˙^−^ + CPY → Intermediates

17 ˙OH/SO_4_˙^−^ + Intermediates → DPS

##### 2.1.3.2. Proposed degradation pathway for CPY *via* OH˙ and SO_4_˙^−^ radicals-based processes

**Scheme 2:**
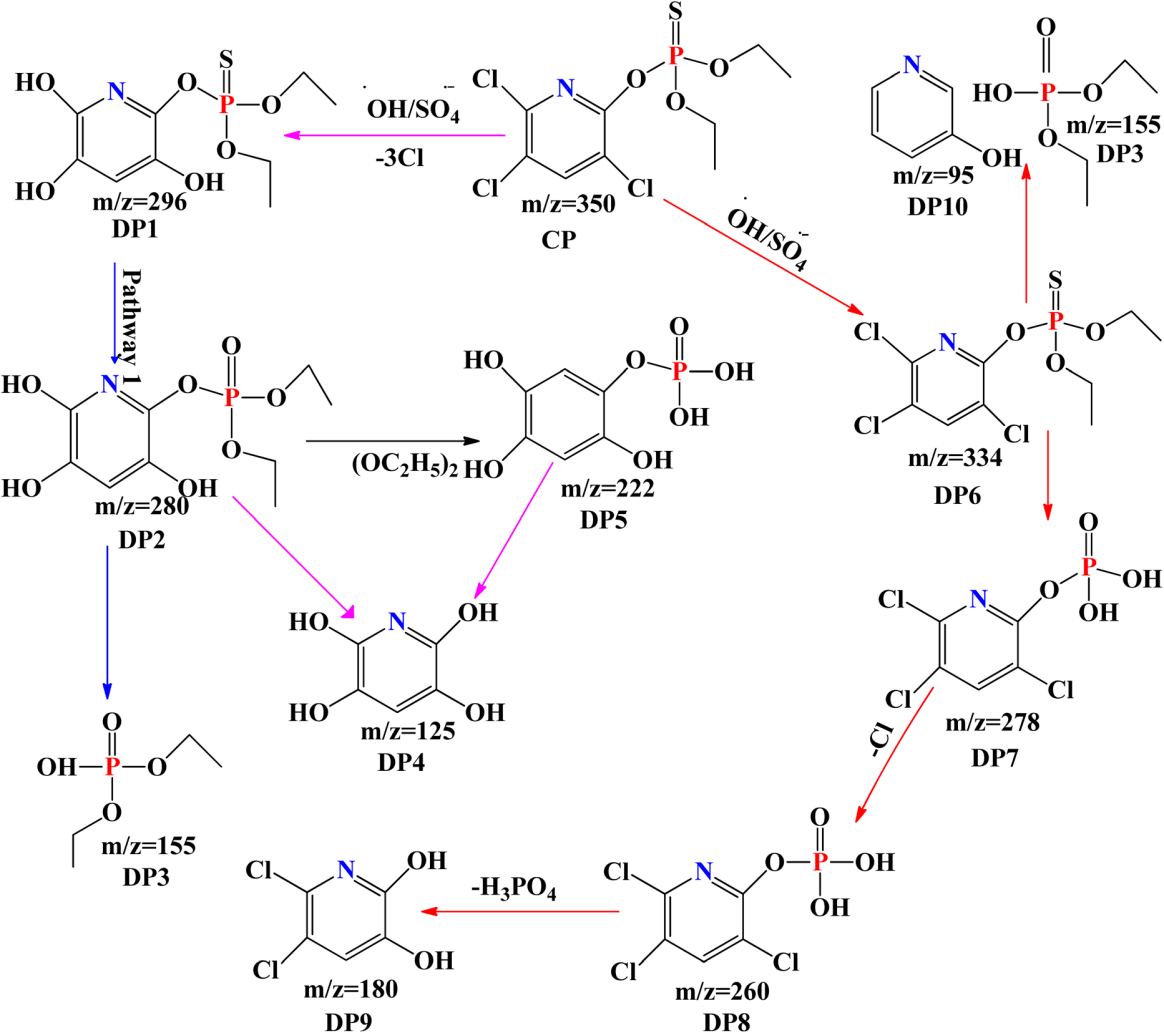
Proposed pathway of CPY degradation by OH˙ and SO_4_˙^−^ based processes.^63^

According to Scheme 2, it was discovered that hydroxyl and sulphate radicals assisted breakdown of CPY generated ten organic compounds all together as indicated in Scheme 2, and two DPs that were inorganic, namely chloride (Cl^−^) and acetate ion (CH_3_COO^−^).^74^ The P–S double bond and the chlorine bonded to the N-containing ring have been identified as the simple locations in the CPY molecule where OH˙ and SO_4_˙ can attack. Through a series of events, the carbon carrying chlorine group was attacked by OH˙ and SO_4_˙^−^, initiating route-1, results in loss of and the formation of hydroxylated product, DP1 as shown in Scheme 1.^74^

The OH˙ and SO_4_˙ continue to attack the DP1, which causes the double bond between phosphorus and sulphur (P=S) bond to oxidize into phosphorus bonded to oxygen through double bond and generate DP2. DP2 undergoes a sequence of reactions that result in bond dissociation between oxygen and carbon and of the Nitrogen-containing ring, generating DP3 and DP4 in the same situation and hydroxylation in the P–O–C region bond, producing DP5.^75^ Moreover, DP5 produces DP4 by losing phosphoric acid. Scheme 2 demonstrates the steps that pathway-II is taken by OH˙ and SO_4_˙ attacking CPY's P=S bond, oxidizing it into a P=O group, and producing DP6. One scenario involves the DP6 hydroxylation and the removal of the R–O–R group, which forms DP7; an additional one entails the bond breakage and removal of the chlorine group among oxygen and carbon (linked to the ring with the N group), which forms DP10 and DP3.^74,75^ After phosphoric acid and chloride were lost through a sequence of intermediary steps, the assault by hydroxyl and sulphate radicals at DP7 resulted in elimination of Cl^−^ and production of hydroxylated compound, DP8, which then underwent additional reactions with reactive radicals to generate DP9.^63^ Effects of some parameter upon degradation efficiency of CYP are discussed below:

##### 2.1.3.3. Effect of [HSO_5_^−^]_0_

Due to the breakdown of CPY by Zn^0^/HSO^−5^ was determined to be critically dependent on hydroxyl and sulphate radical in addition to collision among chlorpyrifos and the catalyst itself, variables that might affect the level of hydroxyl and sulphate radical creation and interactions among chlorpyrifos and zero-valent zinc might have an impact on the elimination of chlorpyrifos. Changing [HSO_5_^−^]_0_ concentration between 10 and 80 mg L^−1^ while maintaining fixed chlorpyrifos and zero-valent zinc levels at 1.0 g L^−1^ and 10 mg L^−1^, accordingly, and a reactor flow speed of 0.2 L min^−1^ proved the initial parameter examined. [Zn^0^/HSO_5_^−^] catalyzed the breakdown of CPY, increasing it from 64 to 99.7% during a 60 minutes reaction interval by increasing [HSO_5_^−^]_0_ from 0.1 to 0.8 mg L^−1^, respectively as shown in Fig. 2(C). The highest breakdown of the desired pollutant at elevated levels [HSO_5_^−^]_0_ was achieved by increasing the level of HSO_5_^−^, according to earlier research by Shah *et al.* (2015), Shah *et al.* (2016), and Sayed *et al.* (2018).^67,71,76,77^

##### 2.1.3.4. Effect of [nZVZn] dosage

[HSO_5_^−^] levels, specifically 10 mg L^−1^ and 40 mg L^−1^, along with the flow speed of 0.4 L min^−1^ shown in Fig. 2(B). When [Zn^0^]_0_ was raised from 0.25 to 2.0 g L^−1^, accordingly, at a 60 minutes interval, breakdown of CPY by Zn^0^ along with the addition of [HSO_5_^−^] rose from 57.01% to 99.23%.^77^ It's possible to link the rise in OH˙ and SO_4_˙ generation frequency to the CPY's increased degrading effectiveness as [Zn^0^]_0_ increases. Raising the concentration of [Zn^0^]_0_ might boost the speed at which OH˙ and SO_4_˙ are formed because nZVZn is the catalyst that breaks down HSO_5_^−^ HSO_5_^−^ producing OH˙ and SO_4_˙.^77^

##### 2.1.3.5. Effect of [CPY]_0_

Thirdly, using a Zn^0^ accelerated HSO_5_^−^ depending on procedure, varying [CPY]_0_ from 2.25 to 25 mg L^−1^ were examined in relation to the catalytic breakdown of CPY. The reactor's flow speed was maintained at 0.2 L min^−1^ and the quantities of Zn^0^ and HSO_5_^−^ were kept fixed at 1.0 g L^−1^ and 40 mg L^−1^, accordingly. When it comes to suggesting parameters for zero-valent zinc, HSO_5_^−^, and rector's flow rate for the excellent elimination of CPY at various levels, the impacts of altering [CPY]_0_ are helpful.

##### 2.1.3.6. Impact of NOM and inorganic ion species

As CPY content is increased from 2.5 to 20 mg L^−1^ at 30 minutes processing periods, the elimination effectiveness of CPY decreases from 90% to 48%, as illustrated in Fig. 2(D). Due to the following factors, CPY's elimination effectiveness is minimal at high [CPY]_0_: (a) by decreasing proportion of hydroxyl and sulphate radicals to chlorpyrifos; (b) greater rivalry to feed active locations on the nZVZn exterior among CPY and its DP's; and (c) increased rivalry among CPY molecules themselves.^71,73,77^

Natural organic matters (NOM) and inorganic ionic species are key components of actual water and have been experimentally shown to have a major impact on the elimination of OH˙ and SO_4_˙ related water contaminants.^78^ Utilizing Zn^0^/HSO_5_^−^ with the additional NOM and ions appeared to have hindered the degradation of CPY, perhaps as a result of rivalry between NOM and ions and adsorption competition on the Zn^0^ surface and CPY for interactions with OH˙ and SO_4_˙ The following order of NO_2_^−^ > CO_3_^2−^ > HCO_3_^−^ > NOM > Cl^−^ > Fe^+2^ > Cu^+^ > NO_3_^−^ was found to be responsible for how ions and NOM impede the catalytic breakdown process of CPY by Zn^0^ facilitated HSO_5_^−^. Owing to its elevated second-order rate constants for hydroxyl and sulphate radicals (as demonstrated in eqn (18)–(20), NO_2_^−^ may effectively fight with chlorpyrifos for hydroxyl and sulphate radical.^63^ By also adding CO_3_^2−^ and HCO_3_^−^ to the reaction solution, the elimination effectiveness of chlorpyrifos by zero-valent zinc facilitated HSO_5_^−^ was decreased to a greater extent. As demonstrated by reactions (eqn (11)–(13), both OH˙ and SO_4_˙ reacts quickly with anions CO_3_^2−^ and HCO_3_ could lower the reactivity.^78,79^ However, even though they had excellent relation with both OH˙ and SO_4_˙ as shown in eqn (17)–(22),Cl^−^, Fe^2+^, Cu^+^ moderately impeded the decomposition of chlorpyrifos by zero-valent zinc catalysed HSO_5_^−^. Chloride ion reacts with hydroxyl and sulphate radical to generate extremely unstable compounds ClOH˙, chlorine and hydroxyl radical, that might facilitate chlorpyrifos breakdown, as demonstrated by reactions.^78^ Reactive radicals and NO_3_^−^NO_3_^−^ have been shown to react slowly eqn (21)–(32), which may be the reason for the minor decrease in CPY degradation rate by Zn^0^/HSO_5_^−^ in the context of NO_3_^−^.^78^

18 ˙OH + NO_2_^−^ → OH^−^ + ˙NO_2_ (*K*_8_ = 8.1 × 10^9^ M^−1^ s^−1^)

19 SO_4_˙^−^ + NO_2_^−^ → SO_4_^2−^ + ˙NO_2_ (*K*_9_ = 8.9 × 10^8^ M^−1^ s^−1^)

20 ˙OH + SO_3_^2−^ → OH^−^ + CO_3_˙^−^ (*K*_10_ = 4.1 × 10^8^ M^−1^ s^−1^)

21 SO_4_˙^−^ + HCO_3_^−^ → SO_4_^2−^ + CO_3_˙^−^ (*K*_11_ = 4.2 × 10^6^ M^−1^ s^−1^)

22 ˙OH + HCO_3_^−^ → CO_3_˙^−^ + H_2_O (*K*_12_ = 8.6 × 10^6^ M^−1^ s^−1^)

23 SO_4_˙^−^ + HCO_3_^−^ → SO_4_^2−^ + CO_3_˙^−^ + H^+^ (*K*_13_ = 8.9 × 10^6^ M^−1^ s^−1^)

24 ˙OH + NOM → products (*K*_14_ = 2.4 × 10^8^ L mol C^−1^ s^−1^)

25 SO_4_˙^−^ + NOM → product (*K*_15_ ≫ 6.0 × 10^6^ L mol C^−1^ s^−1^)

26 ˙OH + Cl^−^ → ClOH˙^−^ (*K*_16_ = 4.30 × 10^9^ M^−1^ s^−1^)

27 SO_4_˙^−^ + Cl^−^ → Cl˙ + SO_4_^2−^ (*K*_17_ = 6.60 × 10^8^ M^−1^ s^−1^)

28 Cl˙ + H_2_O → ˙OH + H^+^ + Cl^−^ (*K*_18_ = 2.1 × 10^5^ M^−1^ s^−1^)

29 ˙OH + Fe^2+^ → FeOH^2+^ (*K*_19_ = 3.20 × 10^8^ M^−1^ s^−1^)

30 SO_4_˙^−^ + Fe^2+^ → FeSO_4_^−^ (*K*_20_ = 9.90 × 10^8^ M^−1^ s^−1^)

31 ˙OH + Cu^+^ → Cu^2+^ + ^−^OH (*K*_21_ = 3.0 × 10^9^ M^−1^ s^−1^)

32 SO_4_˙^−^ + NO_3_^−^ → SO_4_^2−^ + NO_3_˙ (*K*_22_ = 5.0 × 10^4^ M^−1^ s^−1^)

#### 2.1.4. nZVCuC-APS for degradation of *p*-chlorophenol

Zero valent copper (ZVCu) is the metallic form of elemental copper (Cu^0^), which has demonstrated potential in environmental remediation, especially in wastewater treatment. It is a powerful agent for eliminating and lowering a wide range of contaminants due to its strong reactivity and capacity to donate electrons. A wide spectrum of pollutants can be reduced by zero-valent copper (ZVCu), which has a favorable redox potential. The reactivity of ZVCu nanoparticles is increased by their high surface area to volume ratio.^80^ ZVC aids in the breakdown of contaminants by acting as an electron donor in redox processes. Moreover, direct electron transfer (ZVCu) can convert contaminants into less dangerous forms. ZVCu functions as a catalyst in a number of processes, encouraging the breakdown of pollutants, which accounts for its high catalytic activity. Because of its high reactivity, affordability, and ability to remove a variety of contaminants, zero-valent copper is a potential technique for treating wastewater. Its applications will certainly grow and provide issues that need to be addressed by future research and development, making it an essential part of environmentally friendly wastewater governance strategies.^80^

The production of various chemicals in the pharmaceutical, paper, paint, pulp and leather processing, and wood preservation sectors these days uses chlorinated phenolic compounds, which generate a lot of wastewaters worldwide. Due to these substances' low biodegradability and acute and chronic toxicity, they may cause issues with soil, surface, and ground water contamination. All told, effluents from various sources typically contain chlorophenol (CPs), which are classified as extremely hazardous and non-biodegradable environmental pollutants. Many of these primary harmful phenolic compounds, including pentachlorophenol, 2-chlorophenol, *p*-chlorophenol, and 2,4 dichlorophenol, are considered top contaminants by the US Environmental Protection Agency.^81,82^ There are numerous techniques for treating wastewater that can be used to remove or degrade contaminants found in wastewater. A number of biological treatment techniques can break down *p*-CP, but they are limited by high *p*-CP concentrations and extended hydraulic retention times since *p*-CP is poisonous and not biodegradable. Though the biodegradability of organic compounds at polluted waste may be improved by a combination of chemical and biological treatment. To address these issues, recently developed techniques like sonolysis, photo-catalytic oxidation, ozone oxidation, advanced oxidation processes (AOPs) like Fenton and photo-Fenton, and so on are thought to be useful alternative treatment methods for *p*-CP degradation in contaminated water.^83^ Aqueous solutions subjected to sonication create hot areas with high pressure and temperature as cavitation bubbles develop, grow, and burst. To optimize the degrading technique's efficiency, it is also possible to utilize US irradiation in conjunction with oxidants such ozone, hydrogen peroxide, and persulfate (PS). Due to the generation of sulfate radicals, the application of sulfate radical-based AOPs as the degradant agent for a variety of organic compounds has been extensively studied. PS has garnered significant scholarly interest and is frequently employed as an organic pollutants elimination agent due to its higher oxidation–reduction potential (*E*_0_ = 2.01 V) compared to hydrogen peroxide (*E*_0_ = 1.76 V), its excellent water dissolution, non-selectively reactive nature, and affordability.^84^ As a crucial transition metal, copper has garnered significant attention for its ability to produce a range of reactive oxygen species (ROS), such as O_2_^−^˙, H_2_O_2_, and OH˙ These are produced when zero valent copper (ZVCu) induces an alteration in the interaction with molecular oxygen, which in turn diminishes a variety of organic pollutants. In the instance of phenol, 1 mg ml^−1^ Cu^0^ was added as a catalyst and aqueous solutions containing 1 mM phenol and 100 mM H_2_O_2_ were subjected to ultrasound at 520 kHz for sonication. The decomposing effectiveness of phenol increased by 20% and 70% in the ultrasonic and Cu^0^/H_2_O_2_ systems, respectively, after 100 minutes. In contrast to the Fe^+2^/PS and CuO/PS systems (79 and 10%, respectively), the results demonstrated that the Fe^+2^/CuO/PS system could attain a greater degradation level of acetaminophen (92% within 90 min). Furthermore, numerous investigations have confirmed that metal-activated PS works both with and without US irradiation. The study's findings showed that, when compared to ZVCu/PS and nZVI/PS, the nZVCu/PS system was the most efficient way to break down organic materials.^85^ According to published research, two of the main issues with using copper nanoparticles in PS activation systems are rising prices resulting from high copper concentrations and extended reaction times. Thus, it might be necessary to consider a supplementary element.

##### 2.1.4.1. Degradation of *p*-CP in various systems

**Fig. 3:**
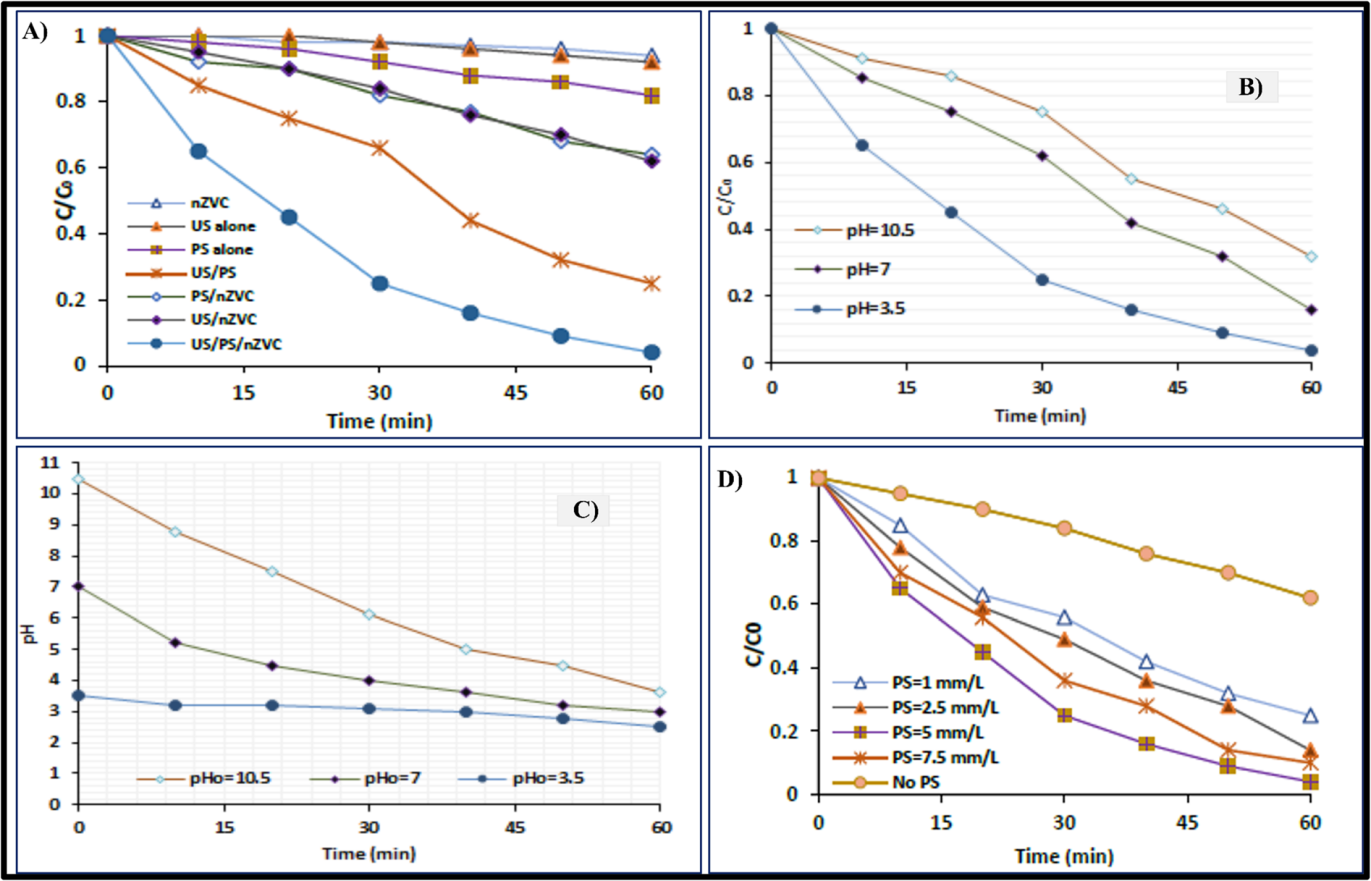
(A) Degradation of *p*-CP under different systems. Experimental conditions:[*p*-CP]_o_ = 50 mg L^−1^, [PS]_o_ = 5 mm L^−1^, [nZVC]_o_ = 25 mg L^−1^, temperature = 20 °C, pH = 3,time = 60 min.^3^ (B) Effect of the initial pH on the *p*-CP degradation rate (a), and pH variation during the reaction (b). Conditions: [*p*-CP] = 50 mg L^−1^, [PS] = 5 mm L^−1^, [nZVC] = 25 mg L^−1^, temperature = 20 °C.^3^ (C) Effect of initial PS concentration on the *p*-CP degradation. Conditions: = [*p*-CP] = 50 mg L^−1^, [pH] = 3, [nZVC] = 25 mg L^−1^, temperature = 20 °C.^3^ (D) Effect of the initial nZVC concentration on the *p*-CP degradation. Conditions: [*p*-CP] = 50 mg L^−1^, [pH] = 3, [PS] = 5 mm L^−1^, temperature = 20 °C.^3^

The degradation of *p*-CP in various systems, such as PS, nZVCuC, US, US/nZVCuC, US/PS, PS/nZVCuC, and US/PS/nZVCuC processes, is compared in Fig. 3(A). The following were the experimental parameters: temperature of 20 °C, pH of 3, time of 60 minutes, ultrasonic intensity of 40 kHz, [*p*-CP]_o_ of 50 mg L^−1^, [PS]_o_ of 5 mm L^−1^, and [nZVCuC]_o_ of 25 mg L^−1^. Fig. 3(A) illustrates the *p*-CP removal rate attained by the two distinct systems, nZVCuC and the US, which could be disregarded. This indicates the low adsorbing capacity of nZVCuC and the little number of hydroxyl radicals produced by the ultrasonic disruption of water alone. Furthermore, the outcomes showed that 5 mm L^−1^ PS could achieve an 18% *p*-CP elimination rate, which was explained by PS's restricted capacity to oxidize. It is evident that the US/nZVCu and PS/nZVCuC systems remove *p*-CP more efficiently than either a single US, nZVCu, or PS system would.^80^

After 90 minutes, the percentage of *p*-CP eliminated for the coupled US/nZVCu and PS/nZVCu system was around 32% and 31%, respectively. According to related results, *p*-CP was resistant to both the oxygen activation generated by ZVC and the sulfate radicals formed when nZVCu activated PS, which was insufficient to keep *p*-CP from degrading. Because of the sulfate radicals produced by eqn (33) and (34), the elimination of *p*-CP in the US/PS system achieved 75%.

33 S_2_O_8_^−2^ + ))) → 2SO_4_˙^−^

34 SO_4_˙^−^ + OH^−^ → SO_4_^−2^

Symbol “)))” represents ultrasonic irradiation and eqn (33), indicated that PS is activated, but this was not a very energy-efficient method. At a 5 mg L^−1^ dosage, the PS/ZVCu system can degrade 2,4 DCP by roughly 82.4%.^86^Eqn (35)–(37) indicate that the combined impact could be characterized as the ultrasound triggering increasing the mass transfer speed of the system and dispersing the accumulation of nZVCu to speed up the corrosion of ZVCu and generate more Cu^2+^ made the PS decompose quicker to create further sulfate and hydroxyl radicals.^87^

35 2CU^0^ + 2H^+^ → 2CU_2_^+^ + H_2_^−^

36 CU_2_^+^ + S_3_O_8_^−^ → SO_4_˙^−^ + CU^3+^

37 SO_4_˙^−^ + H_2_O → SO_4_^3−^ + OH˙ + H_2_^+^

Some parameters are discussed responsible for degradation efficiency of *p*-Cp as below.

##### 2.1.4.2. Effect of pH

The acidic or alkaline reaction media used in AOPs technologies for the breakdown of organic pollutants have an impact. A solution comprising 50 mg L^−1^ of *p*-CP was mixed with 5 mM L^−1^ PS and 25 mg L^−1^ nZVCu to examine the impact of varying starting pH values that vary from 3.5 to 10.5, on the elimination of *p*-CP in the US/PS/nZVCu system. The variations in *p*-CP over time as a function of starting pH are shown in Fig. 3(B) indicates that when the initial pH rises, the breakdown effectiveness of *p*-CP falls. By increasing the starting pH from 3.5 to 7, for instance, the clearance ratio of *p*-CP dropped by 96.3 to 83.5%. In 60 minutes, 68.2% of the *p*-CP had been eliminated as the initial pH rose to 10.5. In line with earlier research,^88^ it is evident that the degree of *p*-CP breakdown increased as the initial pH decreased. With regard to free radicals, acidic circumstances lead to the production of additional sulfate radicals with a redox potential of 2.8–3.1 V, which could account for some of the effective breakdown of *p*-CP in acidic environments.

As per eqn (38), lowering the pH value could facilitate the production of sulfate radicals by PS anion in this particular scenario. Under multiple circumstances, Fig. 3(C) showed the differences in effluent pH with the reaction progression. In acidic, neutral, and alkaline circumstances, as demonstrated in Fig. 3(C), the pH of the solution decreases as the reaction proceeds. Eqn (39) describes how hydroxyl radicals are formed from sulfate radicals by consuming hydroxyl ions in the solution, is what causes this event.^81^

38 S_2_O_8_^2−^ + SO_4_˙^−^ → S_2_O_8_^2−^ + SO_4_^2−^

39 OH^−^ + SO_4_˙ → OH˙ + SO_4_^2−^

##### 2.1.4.3. Effect of persulphate [PS] conc

The impact of an initial PS dosage varying between one to 7.5 mm L^−1^ on the breakdown of *p*-CP using various contact times is displayed in Fig. 3(D). Under consideration the results of different PS concentrations triggered with 25 mg L^−1^ nZVCu in a sample of US. When shown in Fig. 3(D) the *p*-CP degradation rate progressively improved when the PS content rose from 1 to 5 mm L^−1^. The effectiveness of degradation of *p*-CP in 60 minutes was only 75.2% with 1 mm L^−1^ PS, but *p*-CP was nearly completely destroyed with 5 mM PS in 60 above 5 mm L^−1^. According to Monteagudo *et al.*,^85^ this is caused by (a) sulfate radical recombination and a reduction in the amount of oxidant agent to degrade *p*-CP, (b) sulfate radical utilization through reaction with excess PS, and (c) ineffective PS breakdown by hydroxyl radicals that reduces the accessibility of hydroxyl radicals as represented in eqn (40)–(42).

40 SO_4_˙^−^ + SO_4_˙^−^ → S_2_O_8_^2−^

41 SO_4_˙^−^ + S_2_O_8_^2−^ → SO_4_^2−^ + S_2_O_8_^2−^

42 S_2_O_8_^2−^ + OH˙ → OH^−^ + S_2_O_8_^2−^

##### 2.1.4.4 Effect of persulphate [nZVCuC] conc

**Fig. 4:**
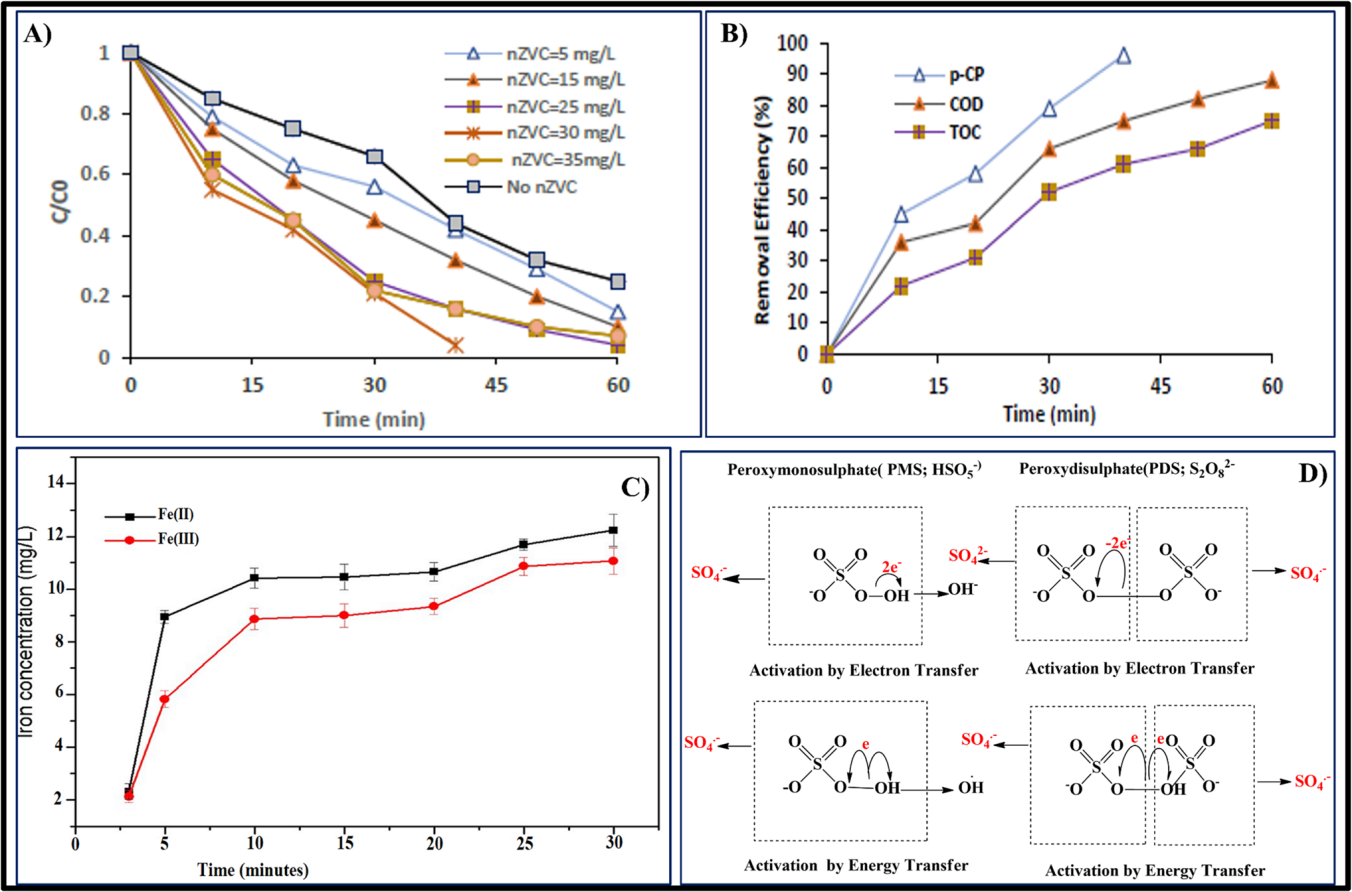
(A) Effect of the initial nZVC concentration on the *p*-CP degradation. Conditions: [*p*-CP] = 50 mg L^−1^, [pH] = 3, [PS] = 5 mm L^−1^, temperature = 20 °C.^92^ (B) TOC, COD and *p*-CP removal efficiency at optimal conditions. Conditions: [*p*-CP] = 50 mg L^−1^, [pH] = 3, [PS] = 5 mm L^−1^ and [nZVC] = 30 mg L^−1^, temperature = 20 °C.^3^ (C) The variations in the concentrations of Fe(ii) and Fe(iii) in the reaction system at various reaction times are shown. Conditions: [ZVI/BC] = 175 mg L^−1^, [atrazine] = 25 mg L^−1^, [PS] = 2.0 mM.^4^ (D) Catalytic mechanism of PMS and PDS through electron and energy transfer processes.^5^

Fig. 4(A and B) shows how the degradation effectiveness of *p*-CP is affected by the initially applied nZVCuC dosage in the US/PS/nZVCuC system, which can range from 5 to 35 mg L^−1^. Degradation speed of *p*-CP can be increased by adding nZVCuC to the US/PS system, as shows. In light of this, the findings suggested that nZVCuC and PS have a certain synergistic impact. When nZVCuC concentrations were 5, 15, and 25 mg L^−1^, respectively, the breaking down percentages of *p*-CP were 84, 88, and 96% post-60 minutes. According to suggestions, after 40 minutes of responses, the *p*-CP degradation effectiveness rose from 76.5 to 97% when the dosage of nZVC was increased from 25 to 30 mg L^−1^. According to the results, 30 mg L^−1^ of nZVCuC is the ideal starting concentration needed to attain the maximum *p*-CP removal effectiveness (97% of *p*-CP removal). This is because a larger initial nZVC dosage produces more active sites and generates a greater quantity of Cu^+^.^80^ Consequently, an increase in sulfate radical production led to an acceleration of PS degradation and an improvement in the rate of *p*-CP elimination. However, a decrease in the *p*-CP removal effectiveness could result from an excess of nZVCuC compared to the ideal level.

Because the system emitted more Cu^+^, a large portion of it might have been absorbed by sulfate radicals that were created, which would have decreased the rate at which *p*-CP was removed, as per eqn (43) and (44). Consequently, in order to maximize the catalytic activity for additional research in this study, a catalyst dosage of 30 mg L^−1^ may be appropriate.

43 CU^+^ + SO_4_˙^−^ → CU^2+^ + SO_4_^2−^

44 CU^+^ + OH˙ → CU^2+^ + OH^−^

##### 2.1.4.5. Mineralization rate of *p*-CP

In order for contaminants to transform into carbon dioxide, water, and other mineral ions, a sufficient quantity of mineralization must take place with the breakdown of organic pollutants. When it comes to treating wastewater polluted with organic contaminants, this procedure is thought to be a workable substitute.^81,84^ The degree of mineralization attained was directly suggested by the TOC and COD contents of the solutions, which were monitored throughout the trials. In ideal conditions, Fig. 4(B and C) illustrates the TOC and COD removal effectiveness of *p*-CP at a starting dosage of 50 mg L^−1^. As illustrated in Fig. 4(B), the degree of mineralization is less than the quantity of organic pollutants that have degraded, which is consistent with the findings reported by Zhou *et al.*^82^ The results indicate that after 30 minutes, the rates of mineralization in regard to COD and TOC removal efficiency were 72% and 58%, respectively. However, under the same operating conditions, the *p*-CP removal rate increased to 96% because it was converted into intermediate metabolites. The elimination effectiveness of COD and TOC extended gradually to 82% and 72%, respectively, when the irradiation period was raised to 60 minutes. Thus, one of the key parameters for the oxidation process is the irradiation times, which influence the photocatalytic mineralization efficiency.^80^

#### 2.1.5. ZVI/BC-PSO for degradation of atrazine

One of the most widely used herbicides with *s*-triazine in agriculture fields was atrazine. This has been widely used for decades throughout the world due to its strong growth-inhibiting characteristics against broadleaf weeds and algae both before and after onset.^89,90^ Atrazine could linger in groundwater and surface waters for a considerable amount of time due to its structural stability, extended leftover period, and not renewable qualities. This would be detrimental to the ecosystem and water quality.^91^ Moreover, because of its carcinogenic and endocrine disrupting effects on a variety of organisms, atrazine has been referred to as an endocrine affecting compound. Thus, atrazine-related pollution needs to receive a lot more scrutiny.^92^ In the past few decades, microbial degradation, precipitation *via* chemicals, electric dialysis, and adsorption have all been used as traditional methods for managing organic contaminants. Advanced Oxidation Processes (AOPs) are a new remediation technology that currently relies on the breakdown of oxidiser to produce OH˙ for pollutants decomposition.^93,94^ The most advanced oxidation methods involve the production of hydroxyl OH˙ to degrade organic pollutants. These methods include O_3_/UV, UV/H_2_O_2_, H_2_O_2_/O_3_, Fenton, Fenton like processes, and photocatalytic breakdown.^95–97^ AOPs based on persulfate (PS) have drawn a lot of attention lately because of their own benefits. PS is regarded as an oxidant with strong oxidizing properties, it can be utilized in advanced oxidation processes to break down a variety of toxic compounds, including *para*-chlorine like, aromatic hydrocarbons having many rings, and 2,4-dinitrotoluene.^89,93^ Zero valent iron (ZVI), which has a tiny particle size, excellent reactivity, and is non-hazardous is extensively employed to catalyse PS to produce redox radicals. It has also demonstrated that ZVI is a PS catalyst that can be employed to remove different types of organic compounds, including sulfamethazine, bis-phenol-A, 2,4-di-chlorophenol, and so forth.^98,99^

##### 2.1.5.1. Effect of BC, ZVI, and ZVI/BC in removing atrazine from PS solution

After a 30 minutes reaction, it was discovered that only 13.66% of the total atrazine had been eliminated from BC by PS treatment. These findings imply that BC showed a modest PS activation, and that atrazine adsorption onto BC was primarily responsible for the trace atrazine removal. Comparably, only 15.24% of the overall sum of atrazine was eliminated during PS treatment solely, which may indicate that PS's capacity for oxidation was constrained in the absence of an activator.^100^ Moreover, atrazine degradation efficiencies were clearly observed to be 23.30% and 73.47%, respectively, at the conclusion regarding the reaction mechanism for ZVI and ZVI/BC activation treatments. Zinc particle accumulation (ZVI) may be the primary cause of the relatively lower activation response of non-supported ZVI, as it can considerably reduce PS activation efficacy.^101^ On the other hand, since ZVI particles accumulate onto BC's porous surface, ZVI/BC can successfully prevent the accumulation of iron particles and improve PS's activation site. Furthermore, ZVI/BC has a considerably greater effect on atrazine degradation in the PS system than ZVI does. The aforementioned investigations clearly demonstrated that ZVI/BC was a more potent PS catalyst for atrazine removal than ZVI.^92^ Establishing safe and effective ways of eliminating atrazine out of ecosystems is essential because of the pollutant poisoning and metabolites, wide distribution, slightly elevated persistence in soil and water, and potential for poisoning to flora. There has been research done on several techniques for removing atrazine from soil and water. Table S3 summarizes research conducted during the past five years in various contexts regarding atrazine breakdown.^102^

##### 2.1.5.2. ZVI/BC-PS system's mechanism of persulfate activation

The variability of iron F(ii), Fe(iii) levels under atrazine elimination conditions the ideal conditions were monitored to notice the PS activation mechanism in the zero-valent iron/biochar-persulphate mechanism as represented in Fig. 4(C).^102^

Quantity of Fe^+2^ and Fe^+3^ in this processes grew progressively as the duration of the reaction grew to 14.24 mg L^−1^ and 12.20 mg L^−1^, respectively, at 30 minutes.^92^ Literature suggests that PS initially oxidized ZVI to Fe^+2^, after that Fe^+2^ might stimulate persulphate to produce sulphate radical, it can be deduced that Fe^+2^ utilization during persulphate activation speeds up ZVI growth and Fe(iii) creation; eqn (45) and (46).^98^ The results above suggest potential mechanisms by which ZVI/BC activates PS. First, in the reaction system, the evenly distributed ZVI on the BC exterior was oxidized to Fe(ii). Fe(ii) that had just been created further stimulated PS to create SO_4_˙^−^, which degraded ATZ. To accomplish the ATZ degradation, ZVI could also simultaneously immediately stimulate PS to generate OH˙; eqn (47). Additionally, the BC_surface_–OH and surface–OOH functional groups on ZVI/BC's exterior might have activated PS through the reaction depicted in eqn (48) and (49) and served as the medium's activator for the transmission of electrons.^103^

45 Fe^0^ + S_2_O_8_^2−^ → Fe^2+^ + SO_4_^−^ + SO_4_^2−^

46 Fe^2+^ + S_2_O_8_^2−^ → Fe^3+^ + SO_4_^2−^ + SO_4_˙^−^

47 Fe^0^ + S_2_O_8_^2−^ + 2H_2_O → 2SO_4_^2−^ + Fe^2+^ + 2HO˙ + 2H^+^

48 BC_surface_–OOH + S_2_O_8_^2−^ → BC_surface_–OO + SO_4_˙^−^ + HSO_4_^−^

49 BC_surface_–OH + S_2_O_8_^2−^ → BC_surface_–O + SO_4_˙^−^ + HSO_4_^−^

The BC_surface_–OH and BC_surface_–COOH groups may be changed into CO˙ and COOCO˙ and COO˙ during the PS stimulation process, which would release a significant amount of SO_4_˙^−^. Lastly, SO_4_˙^−^ would combine with H_2_O to further create OH˙OH˙, which would then degrade atrazine. Therefore, atrazine degradation was successfully promoted by ZVI/BC activating PS.^103^

##### 2.1.5.3. Proposed degradation pathway of atrazine

**Scheme 3:**
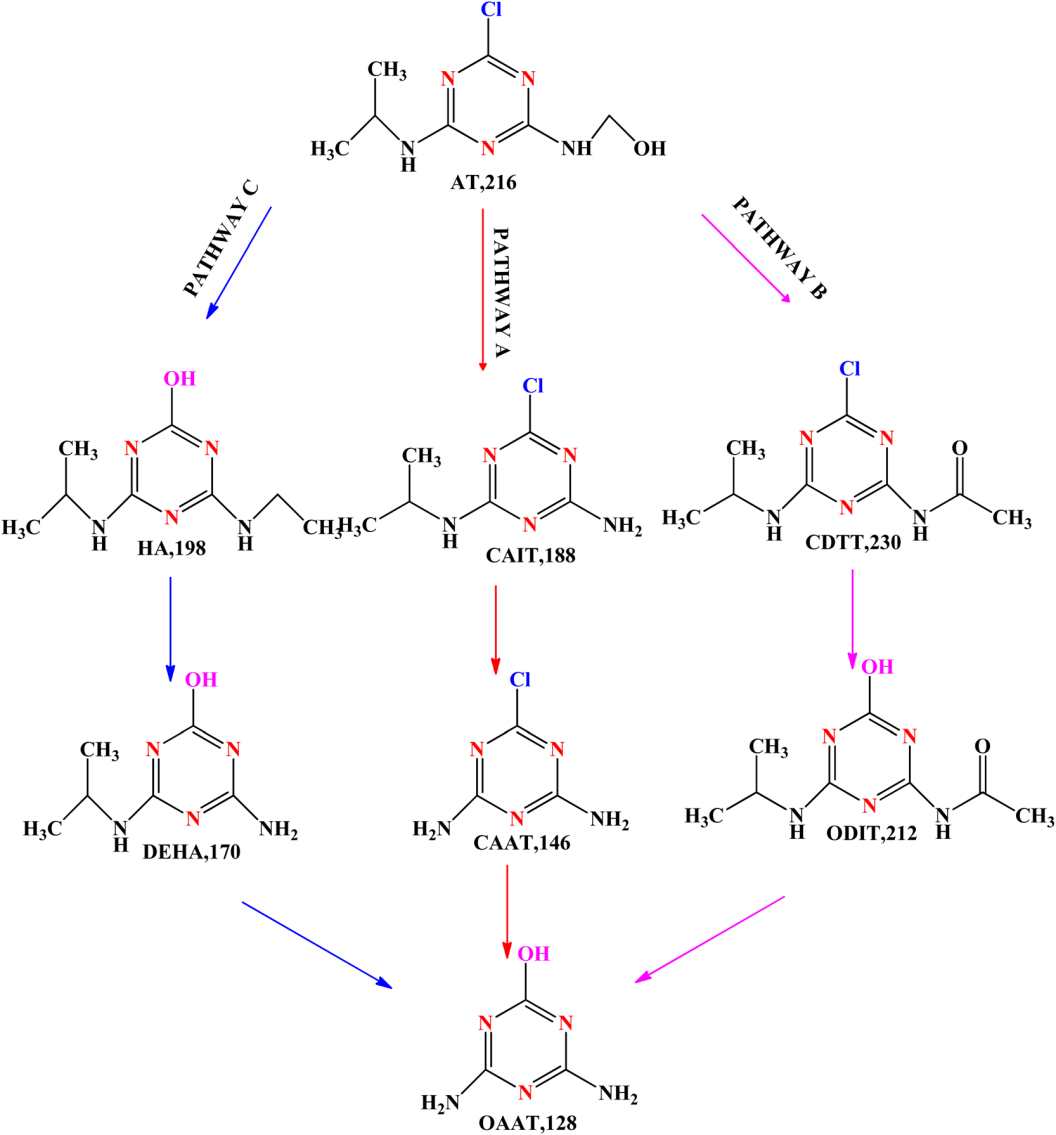
Suggested atrazine degradation pathways in the persulfate (PS)-zero-valent iron and biochar composites (ZVI/BC) system.^92^

Based on relevant literature, three possible processes were postulated to be involved in the degradation pathways of atrazine: dealkylation, alkyl oxidation, and dechlorination–hydroxylation as shown in Scheme 3. Firstly, a dealkylation process leading to the production of CAIT (*m*/*z* = 188) and a subsequent dealkylation process resulting in end compound CAAT (*m*/*z* = 146); this process is primarily caused by electrophilic oxidants SO_4_˙^−^ and OH˙OH˙ in the chemical reaction system, which headed to target the electron-rich areas of ATZ.^89,104–107^ Initially, the solution's oxidizing groups targeted the carbon next to nitrogen through H-abstraction, which led to creation of oxidants with a carbon centre. Then, oxygen that is dissolved could cause carbon-centre radicals to oxidize, producing peroxide radicals O_2_˙, which would then be converted into ATZ-imine by elimination of per hydroxyl radicals (HO_2_˙)^108,109^. Subsequently, *N*-dealkylated derivatives were produced by the hydrolysis of the ATZ-imine group, which subsequently transformed them into OAAT (*m*/*z* = 128). However, the dealkylated products lacked CEAT (*m*/*z* = 174). Demethylation was more prevalent than diisopropylamino, which can be justified by the observation that the hydrogen-atom molar ratio at α-carbon of the ethyl and isopropyl groups is 2 : 1. This indicates that hydrogen-atom in the C_2_H_5_ (ethyl group) is more likely retrieved than in the *n*-C_3_H_7_ (isopropyl group).^90,110^ Pathway B, the second possible degradation pathway, can be defined as follows: atrazine undergoes R-group oxidation, and CDTT (*m*/*z* = 230) that is produced, can then be dechlorinated–hydroxylated to yield ODIT (*m*/*z* = 212). The same peroxide groups that form pathway A may also form the result of alkyl group oxidization CDTT.

These peroxide free radicals could then undergo bimolecular self-terminating decay to generate intermediate tetroxides, which could then break down into ketones or aldehydes. One possible explanation for the formation of CDTT is that OH˙/SO_4_˙^−^ may oxidize intermediate carbinolamine.^92^ Pathway C, the third possible degradation pathway, demonstrated that atrazine generated HA (*m*/*z* = 198) by a dechlorination–hydroxylation process. After that, the HA was dealkylated to create DEHA (*m*/*z* = 170), which was generated by CAIT's dechlorination–hydroxylation. The HO-adduct group started the process of dechlorination hydroxylation of ATZ. This resulted from the instant inclusion of OH˙ to the ipso-place of chlorinated alternative, which produces a germinal chlorohydrin, and the electron movement among atrazine, which generated matching adducts (N-contained free radicals).^94,111^

#### 2.1.6. Impact of novel persulphate oxidation using a nano zerovalent iron system for the degradation of various organic contaminants (TCE, phenol and pyrene)

##### 2.1.6.1. Activation of persulphate through various methods

Typically, oxidants like peroxymonosulphate, also known as PMS, and pyrosulphate (PDS) activate to produce sulphate radicals (SO_4_˙^−^), which have an oxidation–reduction potential of *E*_0_ = 2.5–3.1 V.^112^ In persulfate molecules, the O–O link breaks down, resulting in the generation of radicals that are unstable.^113^ There are currently many ways to activate persulfate, such as activation by heat, UV light triggering, other methods include transition metal stimulation and sonication excitation. One efficient technique is thermal treatment.^114,115^ Molecular O–O bonding in PMS or PDS is broken using thermal energy, which activates persulfate as displayed in Fig. 4(D). Unfortunately, the practical application of heat a activation in wastewater treatment is limited because it necessitates a large amount of energy supply into the reaction system. At the laboratory scale, the degrading of different organic pollutants has been demonstrated by the effectiveness of persulfate activated by UV light. UV radiation having a 254 nm wavelength has been used in most studies because it is readily available and has a high energy productivity. Two important parameters affecting how UV light activates persulfate are the wavelength and UV exposure. Theoretically, sulphate radicals generate more quickly in environments with stronger UV radiation, unfortunately, producing ultraviolet (UV) rays is frequently costly, which restricts the usefulness of UV techniques.^116–118^ Unlike conventional activation techniques, activation by alkali can additionally include the existence of superoxide radicals (O_2_˙^−^) because of the availability of powerful alkaline compounds, as well as to the principal reactive molecules of SO_4_˙^−^ and OH˙. The primary method of activating persulfate in alkaline conditions is the addition of hydroxide. The process of the reaction results in the production of sulphate radicals, it may subsequently produce hydroxyl radicals when it reacts with an alkali.^119^ The process of transition stimulation includes moving one of the transition metal's electrons (such as Fe^2+^, Cu^2+^, Ag^+^, Mn^2+^, Co^2+^, *etc.*) into the molecule of persulfate, the O–O bond breaks, SO_4_˙^−^ is produced.^120–122^ A number of variables, including solution pH, persulfate quantity, and neighbouring anions, may affect how well transition metals activate PS.^123,124^ Activating transition metals is easy, effective, and doesn't need extra energy. Here is the reaction mechanism represented in eqn (50)–(52).

50 M^*n*+^ + S_2_O_8_^2−^ → M^(*n*+1)+^ + SO_4_^−^ + SO_4_^2−^

51 M^*n*+^ + HSO_3_− → M^(*n*+1)+^ + SO_4_^−^ + OH

52 SO_4_^−^ + OH^−^ → SO_4_^2−^ + OH

Nanoscale-sized zero-valent iron particles are referred to as nZVI. Because of their noticeably bigger area of contact, they are more reactive and have superior catalytic performance. ZVI shows a lot of promise as a material for environmental pollution cleaning. A greater percentage of active surface area is provided by nZVI for more effective contact with contaminants than conventional iron fillers. Sulphate radical anions can be produced when nZVI is oxidized to Fe^2+^ and activates persulfate in both anaerobic and aerobic environments. Furthermore, ZVI can combine directly with persulfate to yield SO_4_˙^−^ and OH˙.^120,125,126^ Additionally, the reaction among Fe^0^ and Fe^3+^ can regenerate Fe^2+^ on the nZVI surface, reuse and recycling trivalent iron ions into ferrous ions. Pollutant breakdown and the production of free radicals are encouraged by this regeneration method. The reaction can be shown as follows: eqn (53)–(59).

53 4Fe^0^ + 6H_2_O + 3O_2_ → 4Fe^2+^ + 12OH^−^

54 2Fe^0^ + 2H_2_O + O_2_ → 2Fe^2+^ + 4OH^−^

55 2Fe^0^ + S_2_O_8_^2−^ → Fe^2+^ + 2SO_4_^2−^

56 2Fe^2+^ + S_2_O_8_^2−^ → SO^4^˙^−^ + SO_4_^2−^ + Fe^3+^

57 2Fe^2+^ + S_2_O_8_^2−^ + 2H_2_O → 2SO_4_^2−^ + OH˙ + Fe^2+^ + H^+^

58 SO_4_˙^−^ + 2H_2_O → SO_4_^2−^ + OH˙ + H^+^

59 Fe^0^ + 2Fe^3+^ → 3Fe^2+^

##### 2.1.6.2. Degradation of TCE *via* activate PS/nZVI system through advanced oxidation method

When it comes to breaking down organic pollutants, highly efficient, broadly applicable, and easy to use technique is revolutionary oxidation technique of persulfate activated by the nZVI system. Trichloroethylene (TCE) is highly hazardous compound which can be damaging to manufacturing, human well-being, the natural world, and food hygiene. In order to activate sorbate (PS) for TCE decomposition biochar (BC) was used as a framework for nZVI-Ni dimetallic particles. For oxidation of Trichloroethylene, the nZVI may interact with persulfate to produce free radicals that are reactive (like sulphate radicals).^127^ Enhancing the enzymatic capacity of persulfate, adding nickel Ni to nZVI to create nZVI-Ni alloy, and adding additional active sites and catalyst efficiency can all be achieved by increasing the stability as well as the performance of nZVI. Advance findings shows that newly developed nZVI-Ni@BC composite material has good stability and inhibits nZVI agglomeration efficiently, which improves catalytic activity. TCE elimination efficiency for the nZVI-Ni@BC-persulfate system attained 99% in one hour at 250 mg L^−1^ of nZVI-Ni@BC, 4.0 mM of PS dose, and 3.49 ± 0.55 of pH.^101^ ZVI/BC offers more reaction sites and encourages the production of SO_4_; findings from experiments demonstrate because of the enormous surface area of biochar as a carrier, excellent adsorption efficiency, and sustainability. It took five minutes for the rate of TCE decomposition to exceed 99.4%.

##### 2.1.6.3. Degradation of phenol *via* activate PS/nZVI system through advanced oxidation method

**Fig. 5:**
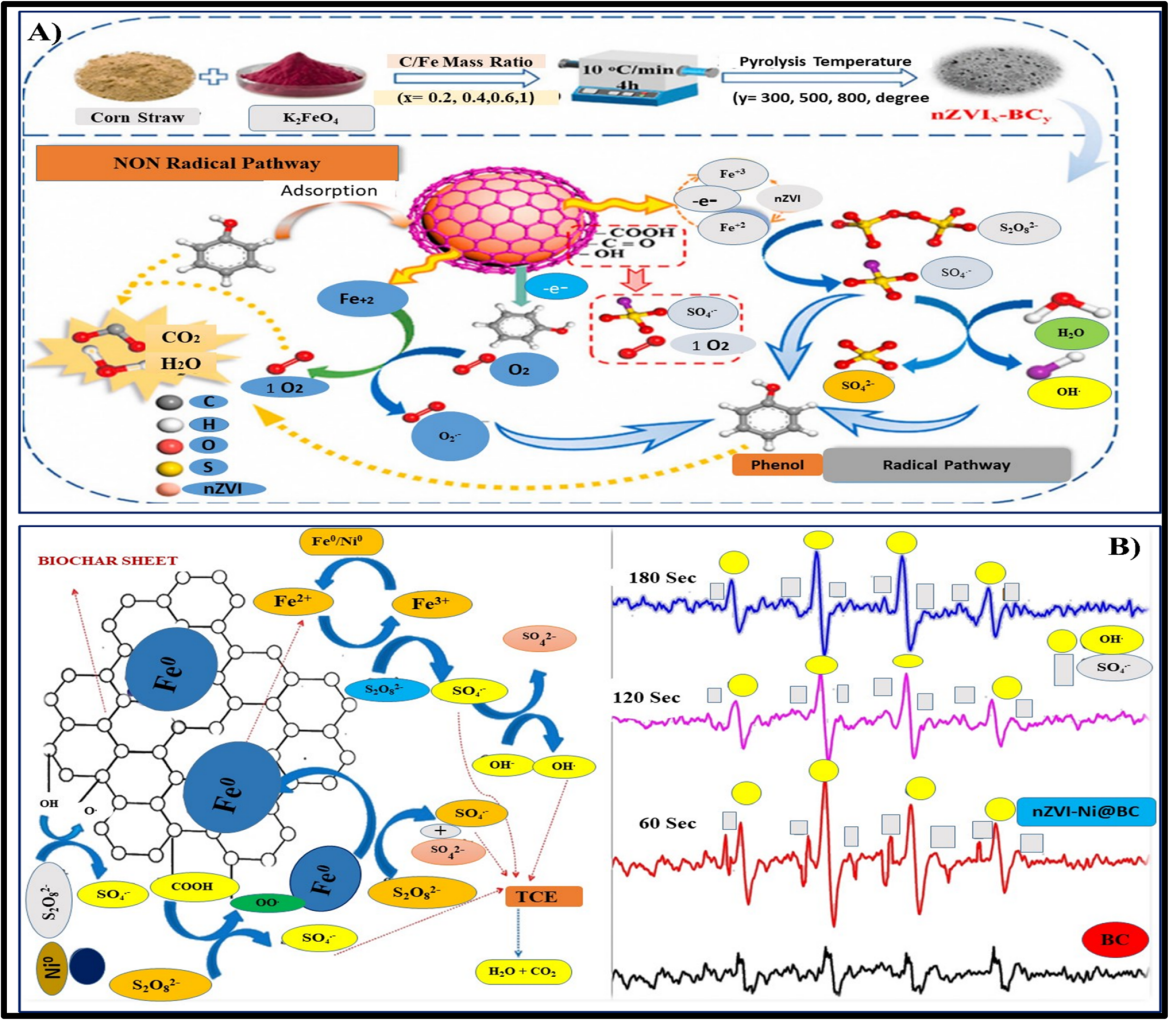
(A) Illustrates the mechanism of phenol degradation using Fe-biochar composite material for activating persulfate.^6^ (B) Degradation of pyrene through activated persulfate with nano zero-valent iron system.^7^

CNS, gastrointestinal tract, breathing system, and other body systems are all at risk from phenol. Anaemia, rashes on the skin, and impairment of the nervous system can all be signs of chronic phenol consumption. Rivers, soil, and the surrounding environment are among the other environmental factors that phenol affects.^128^ Free radical production can be induced by the distinct active sites of iron nanoparticles found in the Fe-BC (iron-biochar) blend and on the exterior of biochar. *Via* direct oxidation, these potent free radicals may degrade the chemical linkages in phenol and convert it into innocuous intermediates. According to experimental findings, the nZVI-BC/PS system demonstrates outstanding performance in various water circumstances and can sustain more than 85% phenol elimination rate after three rounds or fifty days of preservation. In order to stimulate persulfate and concurrently eradicate phenol and Cr(vi) from water containing solutions, Zeng-Hui Diao *et al.* used bentonite-mediated-nano zero-valent iron (B-nZVI). The experimental findings show that the percentages of phenol and the elimination of Cr(vi) in water solution were 71.5% and 99.3%, accordingly.^129^Fig. 5(A) illustrates the mechanism of phenol degradation using Fe-biochar composite material for activating persulfate.^128^

##### 2.1.6.4. Degradation of pyrene *via* activate PS/nZVI system through advanced oxidation method

Major hazards to both human well-being and the natural world exist from the extremely hazardous and cancer-causing polycyclic aromatic hydrocarbon compound pyrene. In a persulfate stimulation system, Junyuan Guo *et al.* employed nZVI-BC to degrade pyrene.^130^ According to the experimental findings, persulfate can cause the nZVI-BC composite substrate to produce a substantial quantity of hydroxyl and sulfate free radicals, which in turn can cause pyrene to undergo an oxidation process and change into safe compounds like fatty acids and small-chain hydrocarbons that are aromatic Fig. 5(B). After an hour of decomposition, the elimination rate of pyrene approached 99.4%, proving that the nZVI-BC-persulfate mechanism operated optimally at pH = 3, nZVI-BC 1.2 g L^−1^, and persulfate levels of 6 mM.^130^

#### 2.1.7. ZVI's for degradation of hexachlorobenzene (HCB) *via* hydrogenolysis

According to the United Nations Stockholm Convention, HCB represents one of the 12 permanent organic contaminants that must be regulated in the initial stage and has a negative impact on both the natural world and the well-being of humans.^131^ Since it has been illegal to manufacture HCB commercially for many years, it is nevertheless generated as an economic residue when various chlorinated naturally occurring solvents and insecticides are synthesized.^132^ Air, soil, and water are only a few of the systems in which is detected. Given that HCB is extremely hazardous, highly bio-accumulative, and persistent in nature, eliminating from soil is a matter of serious worry and a topic of significant scientific and legislative importance.^133,134^ A lot of research has already done in the last few decades to investigate HCB eradication techniques such as microwave remediation, base-catalysed dechlorination,^135^ O_3_-mediated treatments,^136^ photo-catalytic degrading, and microbiological aerosol mineralization.^137,138^

##### 2.1.7.1. Degradation mechanism of hexachlorobenzene

**Scheme 4:**
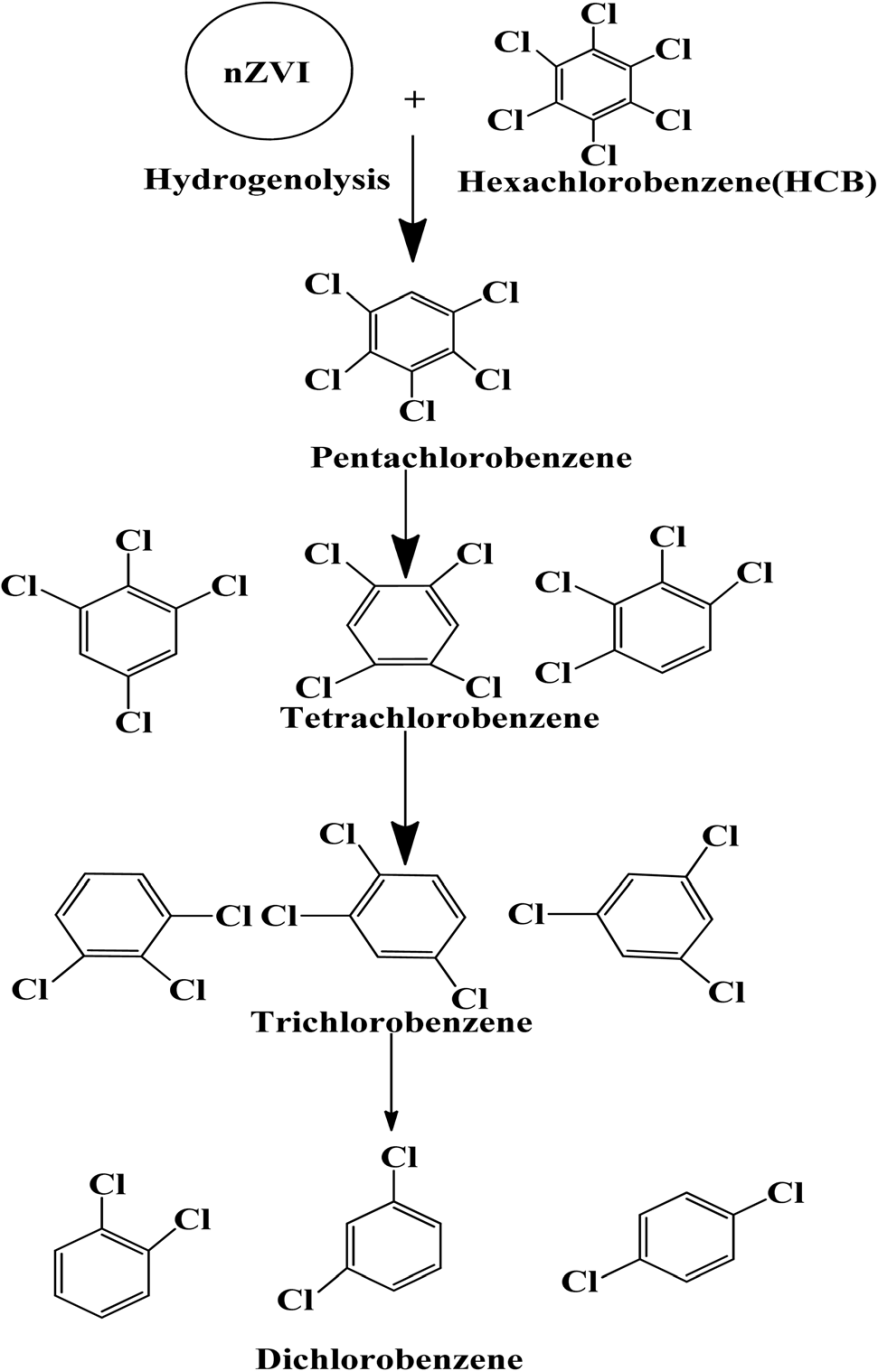
Hexachlorobenzene (HCB) pesticide's hydrogenolysis degradation pathway by ZVI.^141^

It has been observed that zero valent iron nanoparticles (nZVI) can dechlorinate pesticides made from organochlorine compounds like lindane, polychlorinated biphenyls (PCBs), as well as chlorophenol.^139,140^ Two potential dechlorination routes for the breakdown of pesticides by nZVI are hydrogenolysis and α-elimination. The molecular structure of the substances has a significant impact on their chemical reduction process by ZVI. For compounds containing α, β-pairs of chlorine atoms, the primary interaction is the β-elimination. Hydrogenolysis and α-elimination compete for the pollutant degradation of compounds containing chlorine atoms in the α position. On the outermost layer of the tiny nanoparticles the pesticide's C–Cl bond broke while on the reaction, and hydrogen atoms took over the position of chlorine atoms. The hydrogenolysis breakdown mechanism by ZVI of the pesticide hexachlorobenzene (HCB) is displayed in Scheme 4.^141^ Pentachloro benzene was the initial conversion product in the degradation pathway. Following the breakdown of pentachloro benzene, the following intermediates emerged: dichlorobenzene (1,2-DCB, 1,3-DCB, and 1,4-DCB), trichlorobenzene (1,2,3-TCB, 1,2,4-TCB, and 1,3,5-TCB), and tetra chlorobenzene (1,2,3,4-TeCB, 1,2,3,5-TeCB, 1, 2,4,5-TeCB).^142^ The investigation of the chemical breakdown of organochlorine pesticides was conducted in water with ZVI and magnetite (Fe_3_O_4_).^143^ Aldrin, *p*,*p*′-DDT, and lindane were found to have high eradication rates of 79, 81, and 100%, respectively. The energy generated by the breaking of the C–Cl bonding during the chemicals' manufacturing was expected to cause a slight breakdown of the compounds. In contrast to the other molecules where the chlorine was situated in equivalent positions, the quantity of axial chlorine (Cl) in lindane diminished slowly.^144,145^

##### 2.1.7.2. HCB degradation *via* ZVI, a base catalysed reaction

**Scheme 5:**
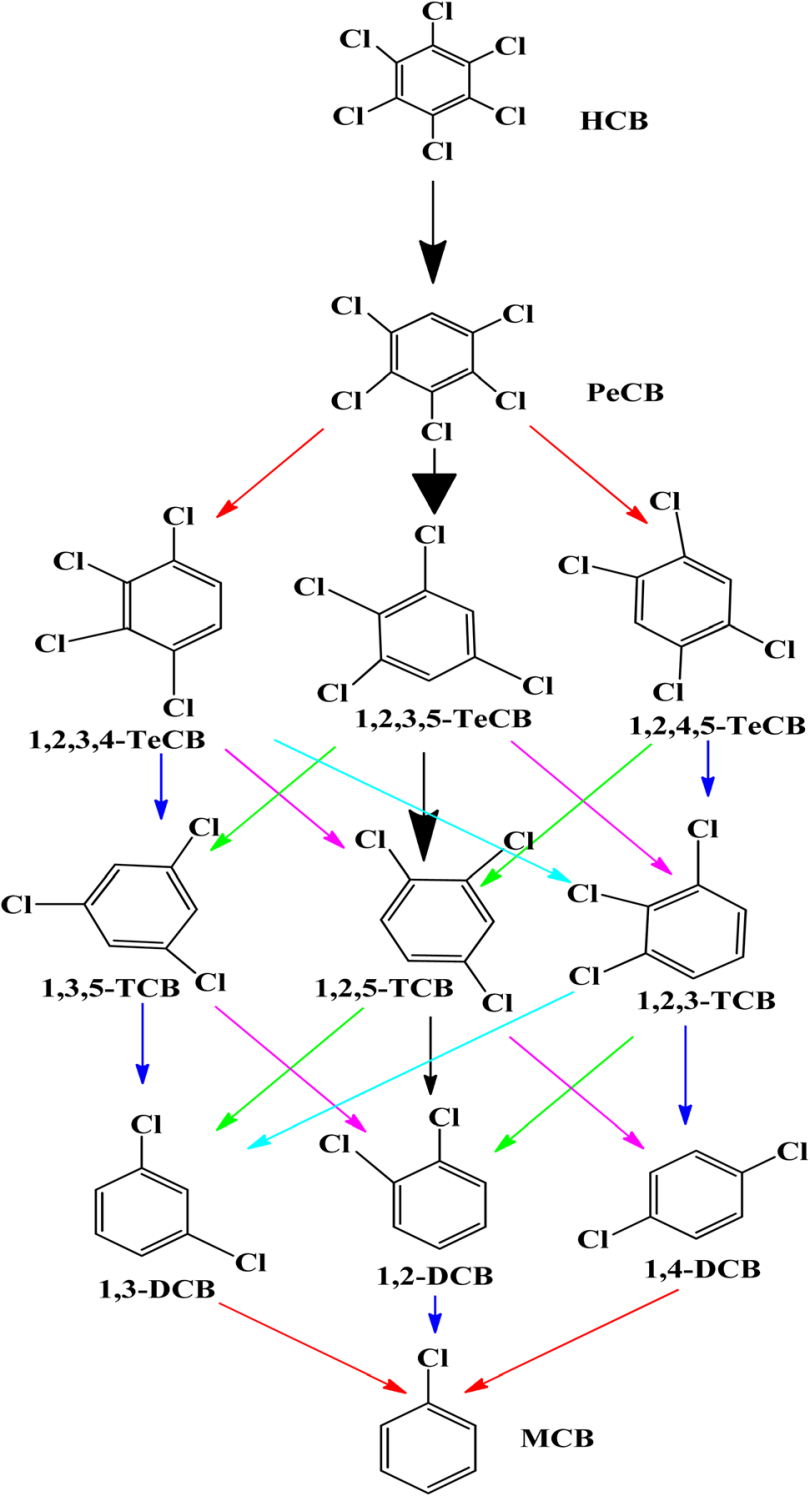
Potential HCB dechlorination routes. The primary HCB dechlorination pathways are indicated by bold arrows.^146^

Each of the detectable isomers of intermediary chlorobenzenes in the chain reaction mechanism, comprising leftovers and paraffin oil, throughout HCB reducing trials. When zero-valent iron was absent, the HCB mol ratio dropped as response time increased.^146^ When zero-valent iron was absent, the HCB mol ratio dropped as the response time increased. Yet as response time increased, the consequences of HCB reduction displayed a variety of tendencies. As the response time progressed, the mole fractions of tetra-chlorobenzene (TeCB) and pentachloro benzene (PeCB) first climbed and subsequently fell.^146^ As the response time increased, the different patterns of HCB and PeCB in an environment of zero-valent iron resembled the ones in a lack of ZVI. After first increasing, the other compounds like TeCB, TCB, and DCB—started to decline. At zero hours, one hour, one hour, and one hour, respectively, were the highest possible mole ratios of PeCB, TeCB, TCB, and DCB. The value of the temperature rose from 0 to 34 minutes, throughout which time HCB changed into PeCB and TeCB. From 0 to 1 hour, PeCB and TeCB were lowered to TCB and DCB. MCB and benzene might've evolved from TCB and DCB around one hour.^147^ Therefore, the reduction of HCB by base catalysis involved a sequential dechlorination procedure. The suggested HCB having zero-valent iron breakdown routes, which originated from the identified dechlorination intermediates. Step-by-step dechlorination constituted the decomposition mechanism. HCB molecules shed one chlorine atom, resulting in the formation of PeCB.^148^ 1,2,3,5-TeCB proved to be the most abundant of each of the three forms of TeCB that were detected throughout the chemical reduction: 1,2,4,5-TeCB, 1,2,3,4-TeCB, and 1,2,3,5-TeCB. 1,2,4-TCB, 1,3,5-TCB, and 1,2,3-TCB are the possible transformations of 1,2,3,5-TeCB.^146^ The amounts of all three isomers declined in the following order: 1,2,4- > 1,2,3- > 1,3,5-TCB. 1,2,4-TCB being dechlorinated to produce 1,2-, 1,3-, and 1,4-DCB as by-products, with MCB becoming the final by-product as displayed in Scheme 5. Generally speaking, the main base-catalysed dechlorination mechanism for HCB using ZVI were HCB → PeCB → 1,2,3,5-TeCB → 1,2,4-TCB → 1,2-DCB → MCB.^146^” The main process by which chloride-containing organic compounds dechlorination is believed to include the exchange of electrons from ZVI to the chlorine molecule.^147,149^ A less effective method than catalytic or transferred electrons, according to several publications, is base activation of PCBs. Initially, the thermal breakdown of the paraffin oil produced hydrogen eqn (60). The procedure also produced additional HC's and C (carbon). Additionally, the base triggered the HCB above a temperature higher (326 °˙C). Eqn (61) indicate that, iron surfaces have the potential to capture HCB and intermediary CB's.^146^60
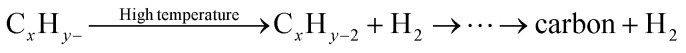
61
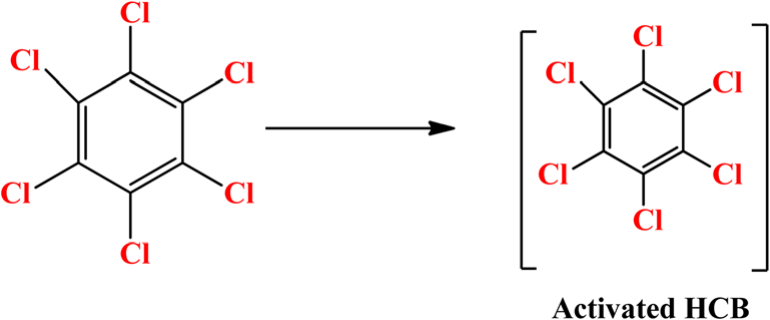
62
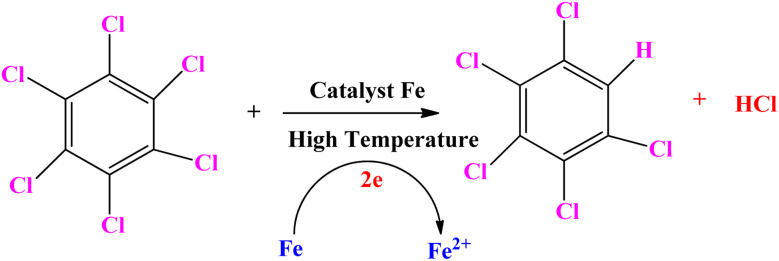


**Fig. 6:**
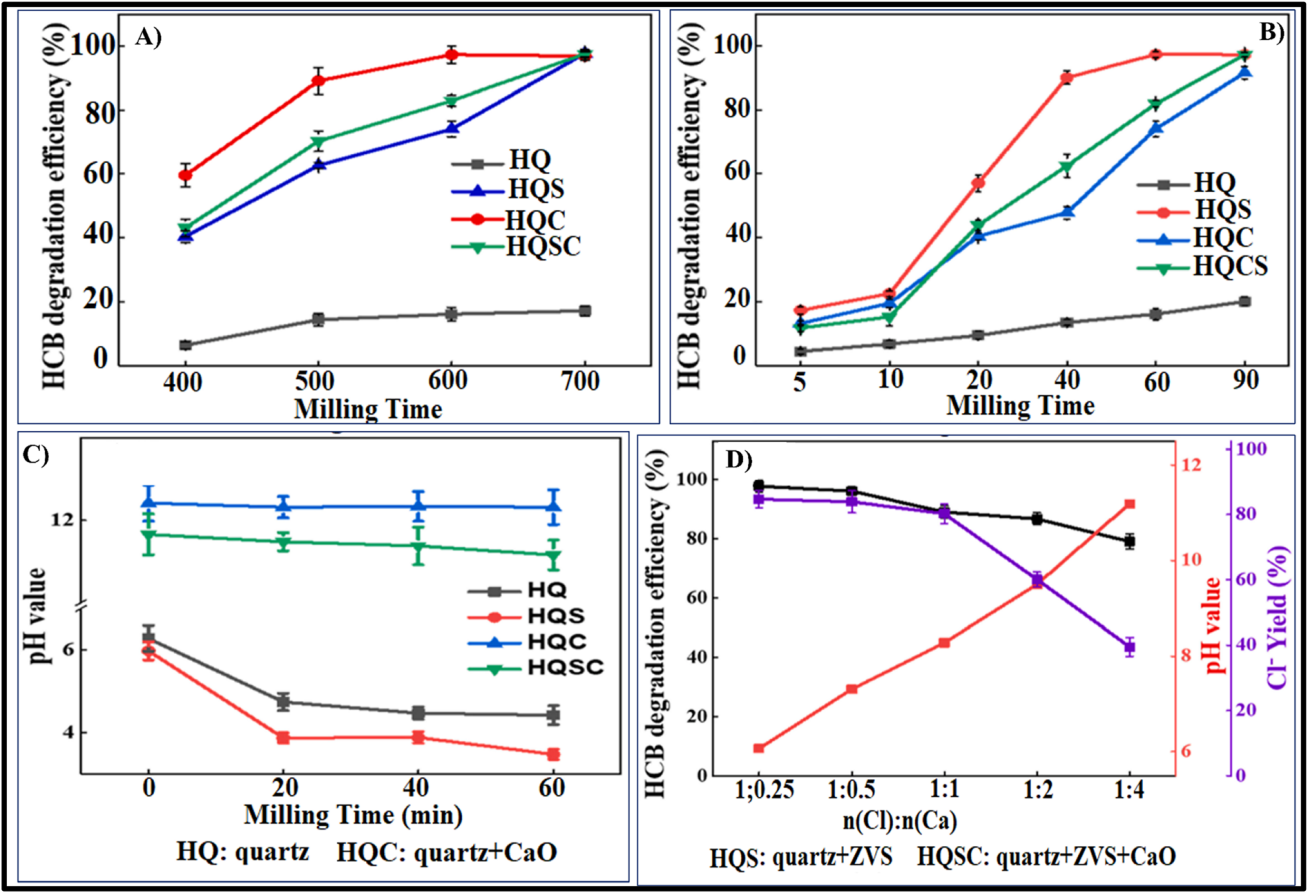
The degradation efficiency of HCB in different additive systems. (A) different milling speed (milling time: 60 min). (B) different milling time (milling speed: 600 rpm). (C) pH at different milling time. (D) HCB degradation rate, dechlorination rate and pH under different Cl/Ca molar ratio in HQSC system (milling at 600 rpm for 60 min).

The removal of chlorine of HCB may be facilitated by ZVI. Dechlorination triggered by water of HCB took place in the context of liberated electrons by iron after the active HCB and hydrogen had travelled to the outermost layer of iron. Hydrogen thus took the place of a single Cl atom in HCB as represented in eqn (62).^146^ A likely ZVS degrading mechanism for HCB is depicted in Fig. 6. Radicals are extensively studied in the mechanochemical breakdown of POPs, and intense milling reveals the newly formed exterior of ZVS to liberate free electrons for HCB chlorine removal. The assault by free electrons on the C–Cl connections, rather than the C–C linking at many locations in HCB, resulted in its breaking and HCB's instability. Then, HCB's benzene ring freed up and broke apart into tiny organic compounds. These little chemical molecules are still receiving the energy that was given to them by free electrons and ultimately degrade to amorphous forms of carbon and graphite. There have been prior reports on this technique for breaking down organic contaminants without the use of an H-donor.^150,151^ ZVS mostly breaks down HCB, HCB → graphite, and amorphous carbon *via* this route. However, humidity is not entirely eradicated, and as a result, zero-valent metals can combine with H_2_O to produce active-H, which would serve as an alternative source of H to feed the process.^152^ Following the splitting of C–Cl, these functional H substituted the location of Cl to generate an entirely novel persistent molecule, which clarifies the identification of PeCBs, TeCBs, and TrCBs as displayed in Fig. 5(B).^153^ Owing to the object's lack of water, there wasn't sufficient functional hydrogen to take the location of Cl in CBs, which decreased the quantity of chlorine removal mediators according to hydrogenation reactions. HCB → PeCB → TeCBs → TrCBs → graphite and amorphous carbon was the particular degrading process.^153^ Additionally, the destiny of the removed chlorine is shown. Initially Cl mixed with ZVS to create SiCl_4_. Next, SiCl_4_ came into touch with the humidity in the vessel and underwent hydrolysis to produce HCl and hydrated silica. Finally, HCl was converted to CaCl_2_ by the addition of CaO, guaranteeing the neutrality of the treated material. Other potential processes included the breakup of HCB and its by-products, which led to the emergence of tiny chlorinated molecules such as CH_2_Cl_2_, CHCl_3_, and CCl_2_=CCl_2_. Even though zero-valent metals, including Fe^0^ and Cu^0^, have good reduction tendency to HCB, uncontrollably generated macromolecular polymers were an inevitable consequence that were disregarded.^154,155^

##### 2.1.7.3. Quantitative analysis degradation of HCB through various milling methods catalysed by ZVSi

Degradation effectiveness of HCB in chemical systems under different settings summarized statistically in Fig. 6(A–D). Grinding variables, such as speed and time, are important elements that significantly influence breakdown performance. No matter how quickly or how long the quartz sand was milled, the HQ system's ability to degrade HCB was restricted. The degrading effectiveness in HQS, HQC, and HQSC systems exhibited a quick increase with an increase in milling frequency as shown in Fig. 6(A) and the duration of milling depicted in Fig. 6(B) as demonstrated by the figures. The chronological progression of the deterioration rate is HQS > HQSC > HQC > HQ, regardless of the milling duration or speed. When it comes to grinding quickness, the HQS system's HCB degrading effectiveness was able to exceed 97.32% at 600 rpm, whereas the HQC and HQSC systems needed to reach above 97% at 700 rpm. When it comes to grinding time, the HQS system could provide a degrading effectiveness of 97.28% at 60 minutes, whereas, the HQC system needed to mill for 90 minutes in order to accomplish a breakdown efficiency of 97.24%. Using distilled water as a dispersant, researchers have also examined the variations in sample solution pH as shown in Fig. 6(C). A *t* milling time following treatment. Because of the inclusion of alkaline CaO, HQC and HQSC mixtures exhibit a pH level in the vicinity of 11.5–12.2. This effect was also noticed in studies involving alkaline solutions (NaOH, KOH, CaO, and Ca(OH)_2_).^156^ The evaluation of various additional systems reveals that ZVS has outstanding HCB degrading capabilities.^153^ Prior studies have indicated that during HCB decomposition, the majority of Cl atoms may be released from the benzene rings. Therefore, understanding the potential form of Cl in the outcome of degradation is crucial for assessing the impact of breakdown and comprehending the degrading mechanism. The formation of SiCl_4_ through the reaction of HCB chlorinated with Si was verified in studies using SiC as an additive as a potential by-product of HCB de-chlorination on ZVS.^157^ Furthermore, because of the high propensity for hydrolysis, SiCl_4_ combines with H_2_O to create HCl. While determining the precise concentration of SiCl_4_ in an ingredient is a challenging task, the pH of the solution can serve as an indicator of quality by tracing the SiCl_4_ transition profile, as seen in eqn (63).^158^

63 SiCl_4_ + 3H_2_O → H_2_SiO_3_ + 4HCl

The main pathway of HCB breakdown was determined to be the dechlorination phase; where the Cl/Ca molar ratio was below 0.5, it was observed that the percentage of dissolved chloride ions might exceed 80%. Nevertheless, when the amount of CaO added increased, the pH value increased even as the production and effectiveness of soluble chloride ions degraded steadily. When the Cl/Ca molar ratio exceeded 1 : 1, the final amount of soluble chloride ions dropped significantly from 80.2% to 39.4%.^159^ This phenomenon differs from earlier research, which suggests that ZVS is a key factor in the breakdown of HCB and that the presence of Basic solutions promotes HCB degradation. The degraded silicon tetrachloride components' acidity may be balanced by the inclusion of CaO, which would have a beneficial impact. Conversely, the existence of CaO has the unfavourable effect of impeding HCB breakdown because of the reduction in ZVS quantity and the outer layer coating of CaO, which both obstruct electron transmission throughout HCB decomposition. In broad terms, excellent outcomes emerged whenever the molar ratio of calcium to chlorine (Ca to Cl) was fixed at 2 : 1: pH = 7.31, 96.12% breakdown rate, and 83.91% chlorine elimination rate as described in Fig. 6(D).^160^

#### 2.1.8. ZVCu and ZVI, a Fenton-like catalysts for imidacloprid degradation

Being able to produce the extremely active hydroxyl radical (OH˙) through eqn (64), Fenton oxidation serves as one of the most efficient techniques for treating polluted wastewater.^161^ Despite the many benefits of the traditional Fenton process, its commercial use remains somewhat restricted due to its drawbacks, which include the need for an acidic environment (pH of approximately 3), the production of an immense quantity of iron sludges, and the costly nature of Fe^+2^ reagents.^162^ The Fenton-like arrangement, which is aided by a variety of metal catalysts, has drawn a lot of research interest as a solution to these issues. Naturally occurring iron minerals like pyrite (Che *et al.* 2011) and chalcopyrite (Labiadh *et al.* 2019) have been the subject of multiple study attempts recently.^163,164^

64 Fe^2+^ + H_2_O_2_ → Fe^3+^ + OH + OH^−^

In addition, zero valent iron (ZVI) initiated by the Fenton-like process has drawn a lot of interest recently. ZVI is unstable and begins to corrode on its exterior as soon as it is employed in a watery solution.^165^ As a result, it may provide Fe ions that trigger the breakdown of H_2_O_2_ as described in eqn (65) and (66).^166^ It promotes the Fenton-like reaction effectively and is a potent reducing agent that can convert ferric to ferrous state at the iron surface (eqn (67)). Many investigators have examined ZVI and found it to be beneficial.^167^

65 Fe^0^ + 2H^+^ → Fe^2+^ + H_2_

66 Fe^0^ + H_2_O_2_ → Fe^2+^ + 2OH^−^

67 Fe^3+^ + Fe^0^ → 3Fe^2+^

According to Ma *et al.* (2018), using ultrasonic treatment in the modified Fenton process can improve copper rusting to some extent. The phase that was liquid consequently produced greater Cu ions, which increased the speed at which norfloxacin was broken down.^168^ According to Zhang *et al.* (2017), in the ZVC/air framework, H_2_O_2_ may partially break apart into O_2_, which might then encourage copper looping by converting Cu^2+^ to Cu^+^. Uniform Fenton-like reaction begins when Cu ions (Cu^+^, Cu^2+^) dissociate in the solution from ZVC interface. Eqn (68)–(72) reveal the primary mechanisms underlying the Fenton-like process that is triggered by Cu ions.^87^68
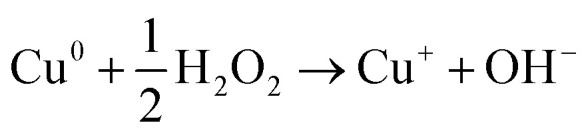
69
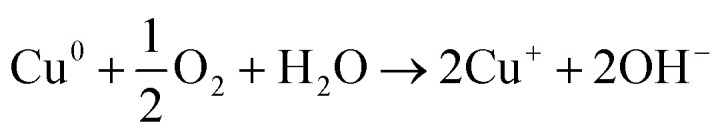

70 Cu^+^ + H_2_O_2_ → Cu^2+^ + OH˙ + OH^−^

71 Cu^2+^ + H_2_O_2_ ⇌ Cu^2+^ + HO_2_˙ + H^+^

72 Cu^2+^ + HO_2_˙ ⇌ Cu^+^ + O_2_ + H^+^

##### 2.1.8.1. Impact of catalyst type on imidacloprid removal effectiveness

**Fig. 7:**
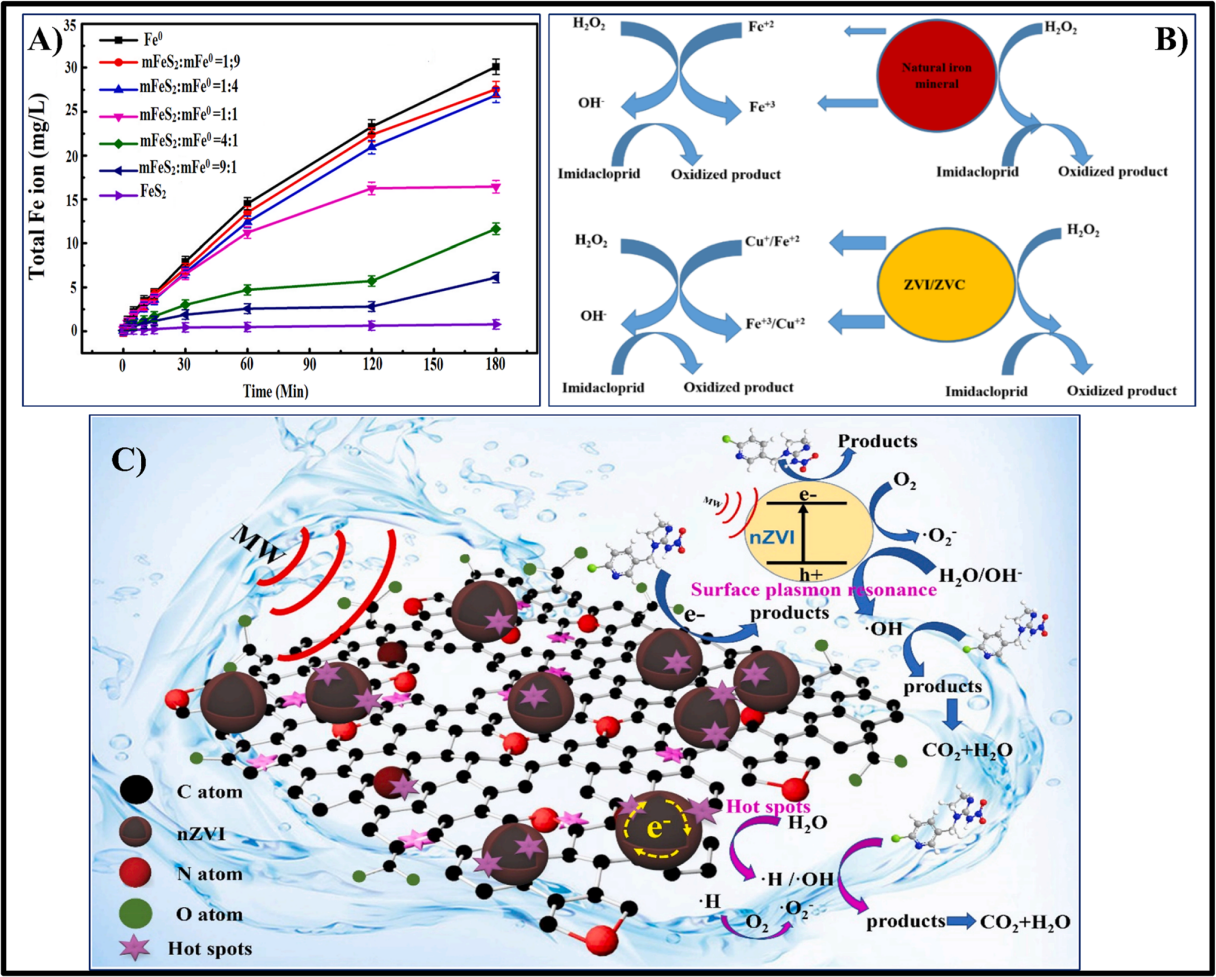
(A) Shows the total Fe ion in various solutions with pH = 4.6, [IMI] = 0.15 momL L^−1^, [FeS_2_/Fe_0_] = 0.1 g L^−1^, and [PS] = 2.5 mmoL L^−1^. (B) Suggested catalytic mechanism for the reaction employing (a) natural iron mineral (pyrite, ilmenite, VTM), (b) zero-valent metal (ZVI, ZVC).^8^ (C) nZVI@N-rGOA/MW's suggested method for removing IMI.^9^

At the starting pH of 3, ZVI had the greatest imidacloprid elimination effectiveness, with an efficacy of 96%, which was substantially greater than the rate of elimination (89%) using Fe_S_O_4_. Considering these circumstances, the least rate of elimination using ZVC was observed. When it comes to mineral catalysts, VTM > ilmenite > pyrite is the order of operation.^161^ The treatments with Fe_S_O_4_, ZVI, VTM, and ilmenite eradicated approximately 79.6%, 73.4%, 48.3%, and 24.2% of COD, correspondingly.^161^ According to the study's findings, there was very little IMI adsorption by ball-milled FeS_2_/Fe^0^, as evidenced by the system's below one percent IMI elimination rate after adding 0.1 mmol L^−1^ PS as displayed in Fig. 7(A). The anticipated increase in the starting PS level was mirrored by the rise in the IMI removal rate. Findings indicated that the removal rate of IMI in the system with a final concentration of 0 mmol L^−1^ PS remained below one percent, suggesting that the IMI adsorption procedure using ball-milled FeS_2_/Fe^0^ proved negligible. It is in the same because a substantial quantity of PS may produce highly energetic radicals which are responsible for the breakdown of IMI, as the eqn (73)–(75) revealed.^169^

73 S_2_O_8_^2−^ + activator → SO_4_˙^−^ + SO_4_^2−^

74 SO_4_˙^−^ + H_2_O → SO_4_^2−^ + OH˙ + H^+^

75 SO_4_˙^−^ + OH^−^ → SO_4_^2−^ + OH˙

It was predicted that the IMI elimination rate would rise as the beginning PS dosage rose. The breakdown of IMI was significantly increased in this investigation by 2.5 mmol L^−1^ of PS; however, an additional increase in PS dosage beyond 2.5 mmol L^−1^ failed to result in a discernible acceleration of the degree of IMI breakdown. This is due to the reality that the quantity of the ball-milled FeS_2_/Fe^0^ would turn into the primary limitation impacting the efficient breakdown of IMI whenever the level of PS exceeded the quantity that might have been triggered by it. IMI breakdown effectiveness and rate were mostly influenced by activating agent's doses and starting PS quantity, indicating that IMI elimination reduced in the absence of sufficient active compound produced by PS and FeS_2_/Fe^0^.^170^

##### 2.1.8.2. Identification of IMI intermediates and the degradation pathways

**Scheme 6:**
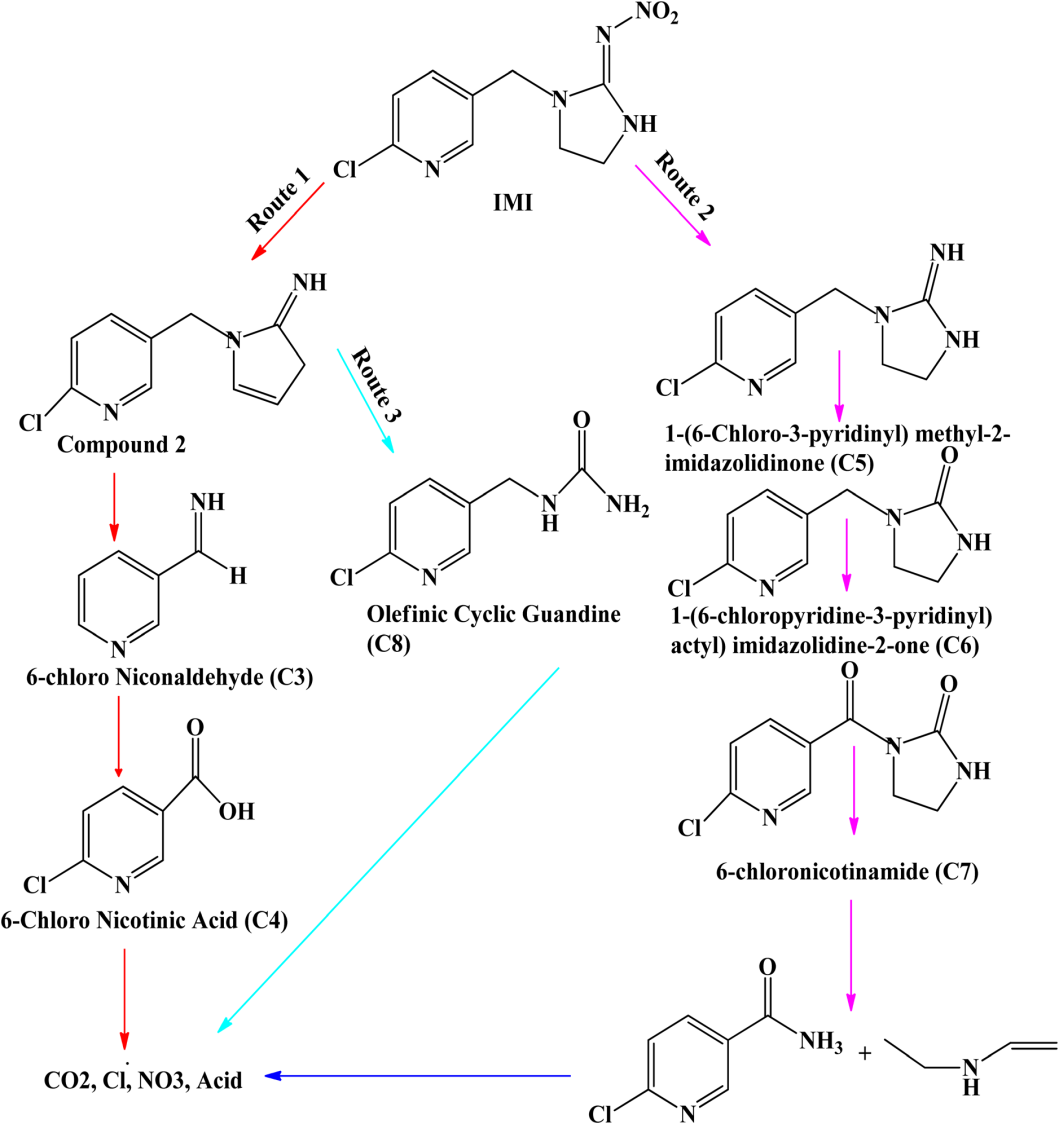
Degradation Mechanism of IMI.^174^

By using GC-MC and LC-MS, reacting homogenous mixtures at various time intervals were examined. Eight by-products were produced, along with their respective analytical properties.^171,172^ Three primary IMI breakdown mechanisms are suggested in Scheme 6. IMI is the peak for the reaction solution at 11.68 minutes before deterioration. Route 1 involves the removal of hydrogen and attacking of ˙OH and SO_4_˙^−^, which breaks the nitrogen–nitrogen bond (IN–N bond) of the nitramino (RNNO_2_) and forms olefinic cyclic guanidine and nitrate. Compound-2 was subsequently broken down into 6-chloronicotinaldehyde, which was subsequently oxidized to 6-chloronicotinic acid through the radical assault on bond C–N, leading to the breaking of rings with five and six members. The primary precursor in the breakdown of IMI was eventually 6-chloronicotinic acid (*R*_t_ = 1.93 min^−1^).^173^ One-(6-chloro-3-pyridinyl) methyl-2-imidazolidinone (C5) is produced in route two concurrently with the elimination of the nitro group and additional oxidation. A subsequent oxidation of compound 5 (C5) results in 1-((6-chloropyridin-3-pyridinyl) acetyl) imidazolidine-2-one (compound 6). This is caused by OH˙ attacking the C-atom across the 5 and 6-membered rings. Then, compound-6 (C6) is attacked by OH˙, resulting in 6-chloronicotinamide (C7). However, compound-2 of route 1 may also result in compound-8, a by-product of the opening ring of olefinic cyclic guanidine, indicating the presence of route 3. CO_2_, Cl^−^, and other inorganic salts might be produced by further oxidation of the acid and some of the intermediaries, indicating that the mineralization of the IMI is completed.

##### 2.1.8.3. Degradation mechanism analysis of imidacloprid under treatment with a Fenton-like reaction system

**Scheme 7:**
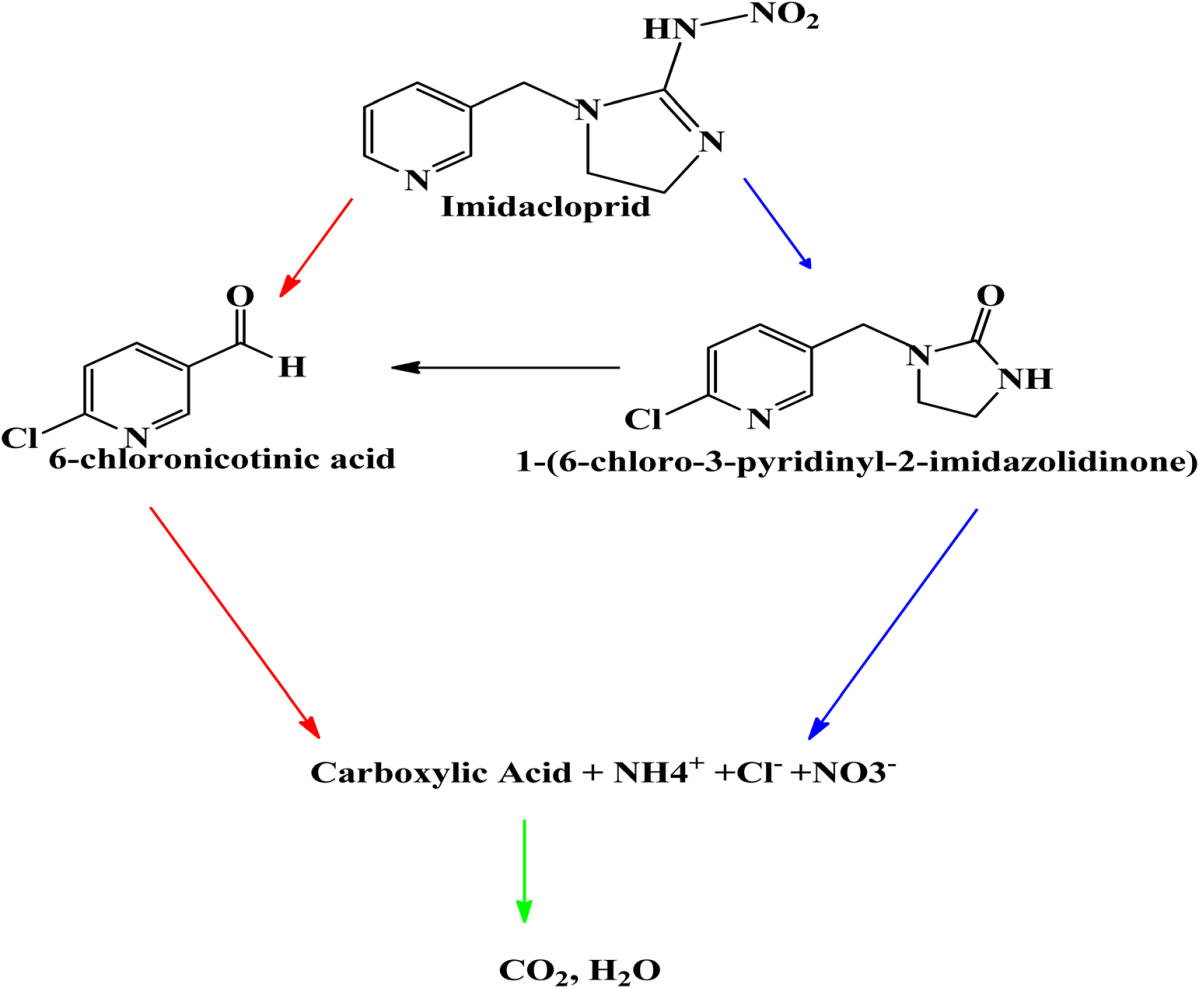
Imidacloprid degradation pathway under treatment with a Fenton-like reaction system.^161^

Regarding which reaction dominates in the heterogeneous Fenton system–the interface reactions on the catalyst interface or the homogeneous reactions brought on by the soluble metal researchers are still at odds of the Fenton-like system. Furthermore, the elimination effectiveness in an acidic environment was significantly higher compared to the alkaline environment, indicating that the pH factor had a major impact on the process of elimination. Both of the aforementioned findings suggest that the mechanism behind the breakdown of naturally occurring minerals triggered by the Fenton-like processes mostly consists of homogeneous reactions brought on by the dissolving phase; the system's participation from heterogeneous interface reactions is less clear.^175^ According to prior research (Kuan *et al.*, 2015; Rezaei and Vione, 2018; Wen *et al.*, 2014; Zhou *et al.*, 2016b) and this experiment, the Fenton-like mechanism accelerated by zero-valent metals is thought to be a homogeneous process linked to absorbed Fe or Cu from rusting of metals displayed in Fig. 7(B).^165,176–178^ However, it is not immediately apparent how heterogeneous interfacial reactions affects the overall system as described in Fig. 7(B). It is important to note that Cu^+^ remained unstable in the solution and in a solution containing water, O_2_ or additional oxidizers will rapidly convert it to Cu^2+^ on a duration of minutes. Nonetheless, given the rapidity of the reaction involving H_2_O_2_ and Cu^+^, it is likely that H_2_O_2_ will be able to gather the temporary intermediate Cu^+^ and include it in the Fenton-like process. A suggested catalytic process is shown in Scheme 7.^179^

##### 2.1.8.4. Degradation of imidacloprid *via* nZVI@N-rGOA

In contaminated water, imidacloprid (IMI) was effectively removed with the help of microwave technology (MW) utilizing a special reduced graphene oxide aerogel doped with nitrogen that accommodated zero-valent iron at the nanoscale (nZVI@N-rGOA).^180^ In less than two minutes, the nZVI@N-rGOA/MW technique practically completely removed the IMI in a 10 mg L^−1^ solution. Beneficial effects, such as pyridinol N positions, accessible pomegranate-like multi-chambered frameworks, and nZVI active centres, enabled MW harvesting, the transfer of mass of the reactants, and the creation of heat, e^−^, h^+^, and ˙OH radicals, leading to the extremely effective MW-based catalytic breakdown of IMI.^180^ Based on evaluations and experimental findings, nZVI@N-rGOA's more effective catalytic efficiency is explained by a MW-driven reaction process. It was observed that nZVI@N-rGOA exhibited a variety of reflections and dispersion of incoming MW, which stopped from leaving the nZVI@N-rGOA, and that it had a 3D pomegranate-like multi-chambered framework made of boundaries with plenty of pores that were visible.^180^ A portion of the incoming microwave radiation (MWs) underwent adsorption and conversion into thermal energy, resulting in the formation of “hotspots” through intrinsic dielectric and conductivity losses of N-rGOA. The remaining MWs developed LSPR, which in turn created electrons (e^−^) and holes (h^+^) by losses from eddy currents of nZVI. The pyridinol-N spots and morphology of the nZVI@N-rGOA were critical for the MW-based catalytic reactions, and N-rGOA significantly increased the nZVI's capacity for MW-harvesting.^180^ Findings indicated that Fe^0^ and iron oxides made up the iron in nZVI@N-rGOA. It was challenging to actually decrease the IMI by passing electrons because of the iron oxides' protection of the nZVI.^180^ It was believed that mechanical warming would trigger the iron oxides to spall and expose the Fe^0^ to long-term exposure, along with improving its catalytic efficiency.^181^ Literature claims that when MW radiotherapy of N-rGOA was applied, it quickly created “hot spots” and restricted overheating surrounding the nZVI due to inherent dielectric and conductivity impairments. The iron oxides spalled as a result of the sudden temperature increase., the freshly revealed Fe^0^ moved electrons to IMI through LSPR, creating Fe^2+^eqn (76), Following its oxidation to Fe^3+^ or the formation of Fe_3_O_4_, Fe_2_O_3_, and FeO eqn (77).

76 MW + Fe^0^ → Fe^2+^ + e^−^

77 Fe^2+^ + H_2_O/O_2_ → Fe_3_O_4_/FeO + H^+^

Effective electron and h^+^ isolation during MW radiotherapy was made possible by the exceptionally large dielectric potential of the iron oxides that were produced (eqn (51)). Following this, the h^+^ and e^−^ in the aqueous environment reacted with H_2_O, OH^−^, and O_2_ to generate powerfully oxidant ˙OH and ˙O_2_^−^eqn (78)–(81). Moreover, the pyridinic N locations, Fe_3_O_4_, Fe_2_O_3_, and FeO created “hot spots” that exploited LSPR and inherent dielectric and conductivity losses to transform the neighbouring H_2_O into ˙OH and ˙H shown in eqn (82).^182,183^

78 MW + Fe_3_O_4_/FeO → h^+^ + e^−^

79 h^+^ + H_2_O → HO˙ + H^+^

80 h^+^ + OH^−^ →˙ OH

81 e^−^ + O^2^ → ˙O^2−^

82 H_2_O + hot spots → ˙OH + ˙H

83 ˙H + O_2_ → ˙O^2−^ + H^+^

Finally, according to eqn (83), the H in the water reacted with the O_2_ to create ˙O_2_^−^. Ultimately, the IMI was metabolized into CO_2_ and H_2_O by these reactive molecules (such as ˙OH, e^−^, and h^+^) having potent reducing and oxidizing capabilities. Fig. 7(C) illustrates potential reaction pathways for nZVI@N-rGOA/MW-mediated IMI elimination.

#### 2.1.9. Nitrobenzene degradation *via* electro-ozonation processes catalysed by NZVI's

An innovative method of treating water (called the E-Fe^0^–O_3_) that combines ozone, micro-size zero valence iron (mZFe^0^), and electrolysis. The integrated technique showed an impressive efficiency in comparison with different control techniques, and it was capable of to remove 90.5% of NB in 20 minutes. Regarding mineralization, the E-Fe^0^–O_3_ process had a greater TOC elimination rate for NB over 120 minutes, but it used fewer kWh than the conventional E-O_3_ and E-Fe^0^ method.^184^ In order to provide an illustration, evaluations were carried out wherein NB was treated in a water-based solution using a combination of E-independently, Fe^0^ solely, ozonation solely in E-Fe^0^, Fe^0^–O_3_, or E-O_3_ procedures. After 20 minutes, electrolysis independently eliminated 17% of the NB, Fe^0^ solely eliminated 18.7%, and ozonation solely eliminated 34.6%. Regarding the binary techniques, NB elimination was 37.1% in the E-Fe^0^ approach and 63.2% in the Fe^0^–O_3_ procedure, which was comparable to the E-O_3_ method (64.7%). On the other hand, the E-Fe^0^–O^3^ procedure showed its greater oxidative capability by eliminating 90.5% of the NB beneath the similar environments.^185,186^

##### 2.1.9.1. Relative contribution to NB removal

There are several ways that the E-Fe^0^–O_3_ mechanism may eliminate NB: direct ozone, electro-direct oxide, ˙OH oxidation, lowering and adsorption of Fe^0^, and ozone generation. In order to assess the respective roles of these pathways in the reported elimination of NB, 20 mM MeOH was introduced into each of each of the processes to eliminate ˙OH. According to Table S7, there was a 2.2% decrease in the Fe^0^ procedure, 1.6% in the direct ozone procedure, and 3.3% in the electro-direct oxidation method. It was 92.8% with OH oxidation, demonstrating unequivocally that ˙OH oxidation predominates over NB elimination in the E-Fe^0^–O_3_ mechanism.

##### 2.1.9.2. Proposed reactions for ˙OH generation

Five pathways are proposed by research for generating ˙OH within the E-Fe^0^–O^3^ methodology: a Fenton-like process, ozone electro-reduction, heterogeneous catalytic ozonation, homogenous catalytic ozonation, and water electrolysis at the anode. Fe^0^'s redox potential is modest (*E*_0_ (Fe^2+^/Fe^0^) = 0.44 V), causing it readily oxidized by ozone, generating a large amount of Fe^2+^, Fe^3+^, and hydroxyl radicals *in situ*eqn (84)–(87).^187^ For 20 minutes, the overall amount of Fe ions throughout the E-Fe^0^–O_3_ pathway increased steadily. Also, O_3_ may be further broken down to generate ˙OH *via* the *in situ* production of Fe^2+^ and Fe^3+^, eqn (88) and (89).^188^ Consequently, the resulting solution is experiencing homogeneous catalytic ozonation.

84 Fe^0^ + 2O_3_ → Fe^2+^ + 2O_3_

85 Fe^2+^ + O_3_ → Fe^3+^ + O_3_˙^−^

86 H^+^ + O_3_˙^−^ → HO_3_ → ˙OH + O_2_

87 Fe^2+^ + O_3_ → (FeO)^2+^ + O_2_

88 (FeO)^2+^ + H_2_O → Fe^3+^ + ˙OH + OH^−^

89 Fe^3+^ + O_3_ → (FeO)^2+^ + ˙OH + O_2_ + H^+^

Reactions; A: oxidation by OH; B: electro direct oxidation; C: direct ozonation; D: the reduction and adsorption of Fe^0^; MeOH represents the certain concentration of methanol; E-Fe^0^–O_3_(MeOH) represents the presence of MeOH in the processes; electrolysis (MeOH) represents the presence MeOH in electrolysis processes; ozonation (MeOH) represents the presence of MeOH in ozonation.^184^

To produce significant heterogeneous catalytic ozonation. The primary locations responsible for ozone conversion are the ˙OH on the surfaces of the various catalysts, and the solution pH is one of the important factor affecting catalytic activity (eqn (68)–(70)).^189^ The iron oxide surfaces' protonated hydroxyl groups may react with ozone molecules, gradually converting them into ˙OH, eqn (90)–(96).^190^ In order to destroy absorbed contaminants, the surface-adsorbed OH may either permeate into the majority of solution or oxidize adsorbed contaminants *in situ*.

The following were the steps of reaction:

90 FeO–OH + H^+^ ↔ FeO–OH_2_^+^

91 FeO–OH + OH^−^ → FeO–O^−^ + H_2_O

92 FeO–OH_2_^+^ + O_3_ → FeO–OH˙^+^ + HO_3_˙

93 HO_3_˙ → ˙OH + O_2_

94 FeO–OH˙^+^ + H_2_O → FeO–OH_2_^+^ + ˙OH

95 FeO–OH + 2O_3_→FeO–O˙^−^ + HO_3_˙ + O_2_

96 FeO–O_2_˙^−^+O_3_+H_2_O→FeO–OH+2O_2_ + HO_2_˙

Concerning the electro-reduction of O_3_, it has been already reported before (Zhan *et al.*, 2019; Wang *et al.*, 2020) that a portion of O_3_ is reduced immediately in a system consisting of three electrodes at the cathode area and then converted to ˙OH, as described in eqn (97) and (98).^191,192^

97 O_3_ + e^−^ → O_3_˙^−^

98 O_3_˙^−^ + H_2_O → ˙OH + O_2_ + OH^−^

According to the ozonation catalysis process, oxygen may be created throughout the procedure, which relates to the Fenton-like reaction. A portion will additionally dissolve in the solution after entering from the ozone producer. The Fenton process can precipitate the production of ˙OH in an acidic condition by reducing oxygen to H_2_O_2_, as described in eqn (99)–(102).^193^

99 Fe^0^ + O_2_ + 2H^+^ → Fe^2+^ + H_2_O_2_

100 Fe^2+^ + H_2_O_2_ + H^+^ → Fe^3+^ + ˙OH + H_2_O

101 Fe^2+^ + H_2_O_2_ → Fe^3+^ + ˙OH + OH^−^

102 Fe^3+^ + e^−^(cathode−area) → Fe^2+^

Additionally, the H_2_O_2_ and soluble O_3_ can combine to produce hydroxyl radical, as shown in eqn (103)–(106).^185^

103 H_2_O_2_ + 2O_3_ → 2˙OH + 3O_2_

104 H_2_O_2_ ↔ HO_2_^−^ + H^+^

105 HO_2_^−^ + O_3_ → HO_2_˙ + O_3_˙^−^

106 H^+^ + O_3_˙^−^ ↔ ˙OH + O_2_

Finally, water electrolysis at the anode needs to be taken into consideration. On the anode's exterior, which would immediately generate ˙OH, eqn (107) and (108).

107 M−anode_surface + H_2_O → M(˙OH) + H^+^ + e^−^

108 M(˙OH) → MO + H^+^ + e^−^

##### 2.1.9.3. Reduction of NB by SiO_2_-coated nZVI

**Fig. 8:**
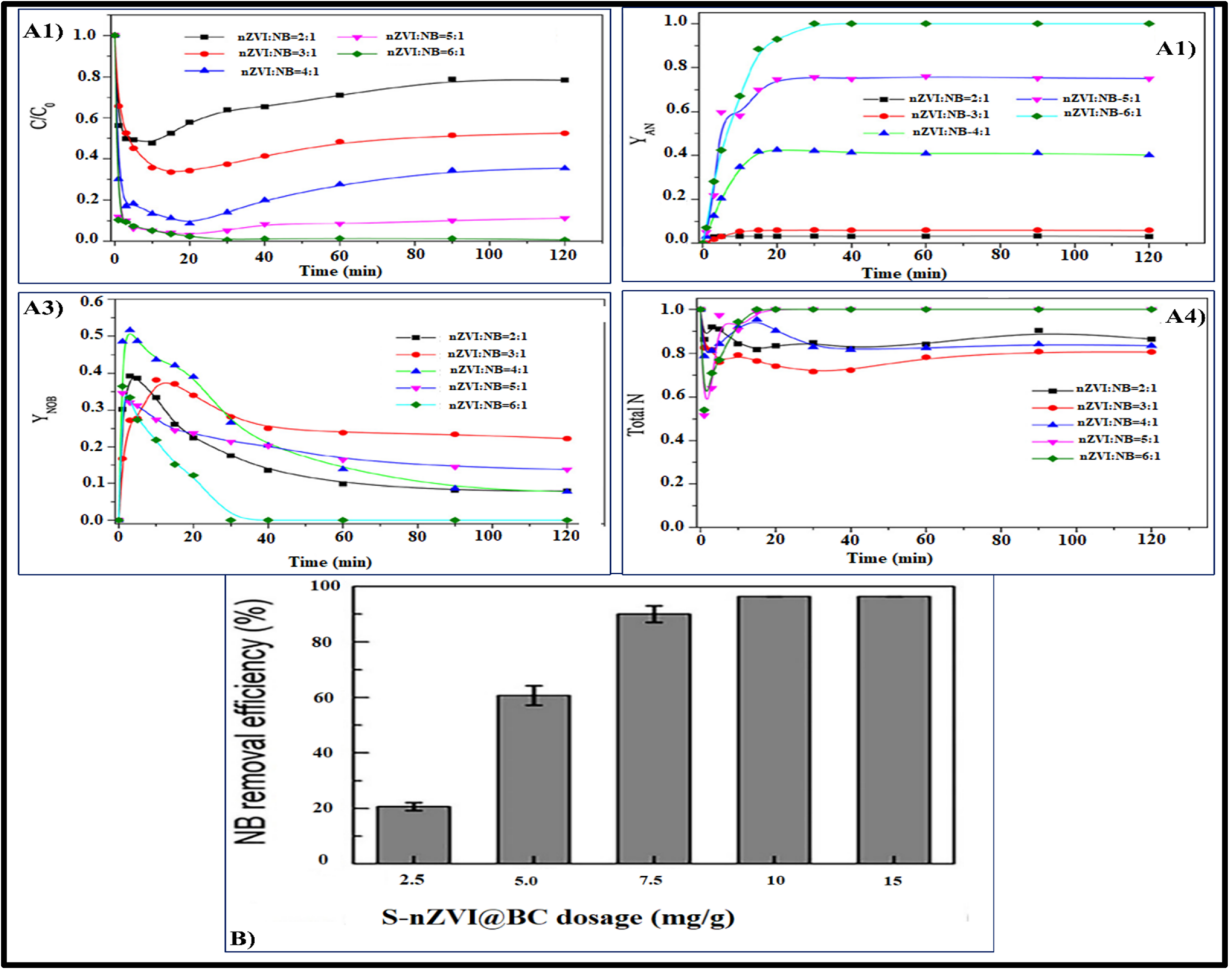
(A1–A4) The reduction efficiency of NB by SiO_2_-coated nZVI. Experimental condition: initial NB = 80 mg L^−1^, the molar ratio of Fe° to NB was 3 : 1, pH = 7.0 ± 0.5, room temperature.^10^ (B)Effect of S-nZVI@BC dosage on the NB removal efficiency.^11^

**Scheme 8:**
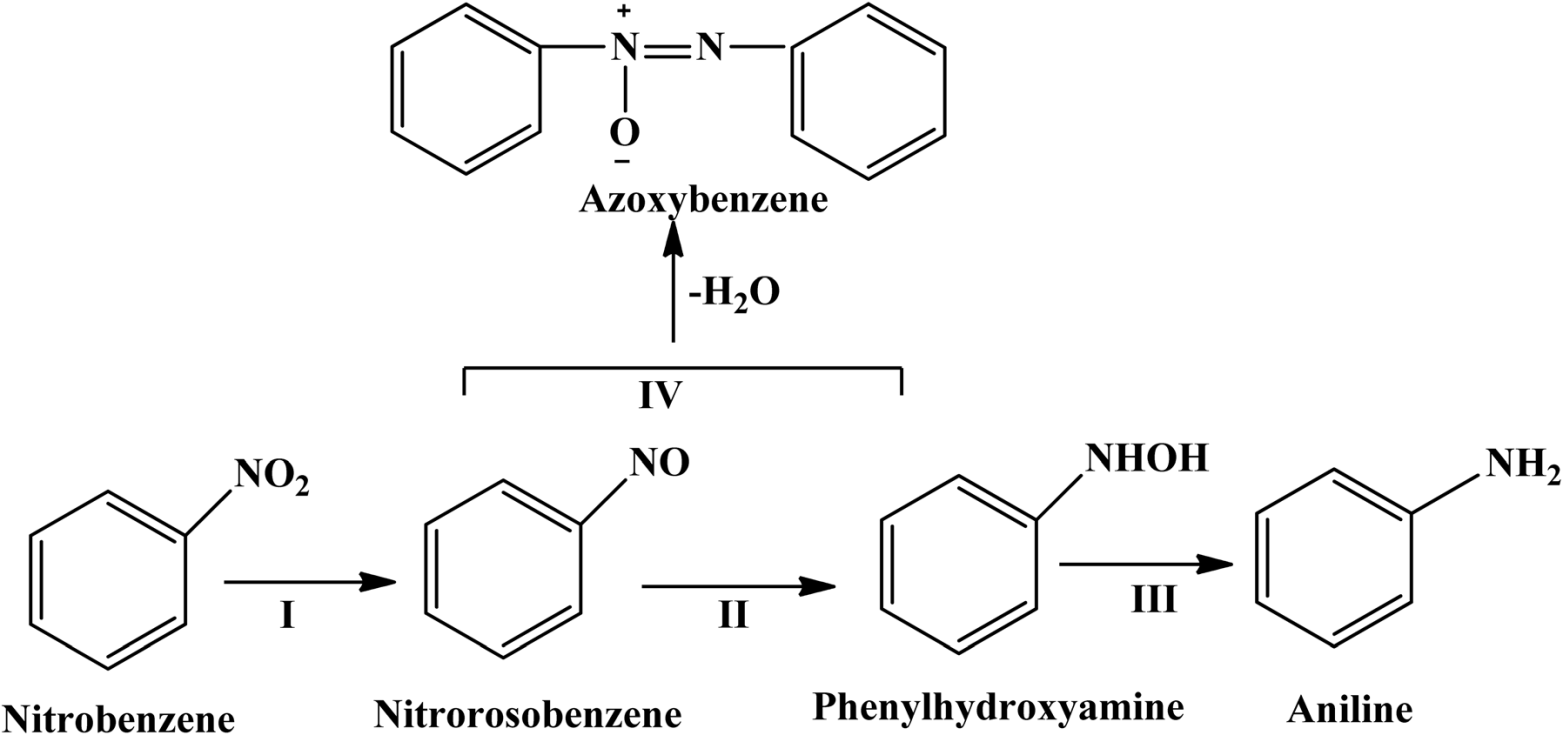
Proposed pathway of NB reduction by SiO_2_-coated nZVI Composites.^194^

An investigation was conducted into the reduction effectiveness of aquatic NB by SiO_2_-coated nZVI. The reduction effectiveness of NB was related to the molar ratio of Fe^0^ to NB (nFe/nNB) and it might have reduced quickly over the initial 15–20 minutes. In accordance, AN was produced and in 30 minutes it achieved equilibrium as shown in Fig. 8(A). With a rise in nFe/nNB, the generation effectiveness of AN also got enhanced as displayed in Fig. 8(B).^194^ NB is capable of being transformed to AN completely whenever the ratio of Fe to NB increases to 6 : 1, and this was the optimum amount of Fe^0^ for reducing the concentration of 80 mg L^−1^ NB. The most common phase in the reduction procedure was nitroso benzene (NOB), which peaked at an intensity of 5 minutes, then steadily dropped and finally reduced to AN in just 30 minutes.^194^ On the other hand, the NB reduction effectiveness increased and then decreased when nFe/nNB remained below 6 : 1, and the NOB production effectiveness showed a similar fluctuation pattern as shown in Fig. 8(C). In contrast to SiO_2_-coated nZVI composites, the ultimate transformation effectiveness of NB to AN only reached 26.6% for bare nZVI, despite the fact the reduction effectiveness of NB was able to exceed 94.2%.^195^ More than fifty percent of the NB was formed efficiently, and the majority was transformed to NOB. The primary cause of this is the porosity of SiO_2_ covering, which can improve the mass transfer effectiveness by facilitating absorbed NB interaction through the internally activated nZVI.^196^ Furthermore, through six consecutive breakdown phases as shown in Scheme 8, the NB reduction effectiveness by SiO_2_-coated nZVI might rise up to 84.7%, according to the reliability and persistence analysis, suggesting that it is a viable option for *in situ* clean-up of groundwater pollution.^197^Fig. 8(D), findings demonstrate the overall mass equilibrium of the entire reduction process, which is expressed as total *N*% in eq. below.Total *N*% = (*C*_NB,*t*_ + *C*_NOB,*t*_ + *C*_AN,*t*_)/*C*_NB_where CNB represents the starting quantity of NB (mmol L^−1^), and *C*_NOB,*t*_, *C*_AN,*t*_, and C_NB,*t*_ represents the amounts of NB, NOB, and AN (mmol L^−1^) at any given point in time (min), accordingly. There may have been more reaction intermediates because the mass balance appeared to indicate a repeatable loss in the first stage (0–10 min). It was comparable to the earlier report.^14^ The overall *N*% might attain equilibrium in 20 minutes if the nFe/nNB increased to a ratio of up to 5 : 1. Nevertheless, even after 180 minutes, the total *N*% could not achieve balance if the nFe/nNB became beneath 5 : 1. These mass balance deficits suggested that the unknown intermediaries might contain certain coupling products. Which weren't important in the synthesis of AN and thus were not likely to be oxidized to NB.^198^

##### 2.1.9.4. Effective elimination of nitrobenzene *via* nZVI/BC

Because of its instability, facile accumulation, and iron leaching, nanoscale zero-valent iron (nZVI) is not frequently utilized in the rehabilitation of polluted groundwater or sewage. This problem was solved by dispersing nZVI over biochar (BC), which produced the nZVI/BC nanocomposite, which greatly reduced nitrobenzene (NB).^199^ But because of the strong magnetic attraction between the components and their elevated energy levels on the surface, the main problems with nZVI are significant clumping and rapid oxidation in the atmosphere, which restricts the applicability.^200^ As a potentially effective substance for environmental clean-up, biochar (BC) is currently attracting attention. Carbon-rich feedstock or other kinds of solid waste are pyrolyzed at inexpensive prices and with little oxygen to create BC.^201^ Engineered NP's may be stabilized and dispersed using BC because of its huge particular surface area, extensive porosity, strong power, and plenty of functional groups on the exterior.^202^ It is therefore a good dispersion medium for nZVI. The adaptable qualities of BC enabling nZVI, (nZVI/BC) in the elimination of pollution might be expected.^203^ BC's excellent adsorption capabilities regarding organic pollutants are partially restored by an abundance of functional groups that contain oxygen, for instance, carboxyl (–COOH) and hydroxyl (–OH). However, nZVI is quite effective in cleaning up pollutants by reducing them upon adsorption.^204^

##### 2.1.9.5. Removal of NB *via* reduction or adsorption

ZVI serves as a reducer and BC serves as an adsorbent in the process of reductive elimination. At the BC surface, impurities primarily sorb, then with ZVI, they decrease. While declining at a slower rate compared to ZVI, BC also leads to decrease. Nitro contaminants are removed by reductive means.^199^ nZVI/BC promoted rapid NB deposition on BC and deposited NB degradation to AN. NB reduction caused BC buffered nZVI to undergo oxidation to Fe_3_O_4_. Its deposition on BC, as opposed to nZVI solely improved NB adsorption and reduction in addition to improving stability and oxidation resistance.

109 C_6_H_5_NO_2_ + 3Fe^0^ + 6H^+^ → C_6_H_5_NH_2_ + 3Fe^2+^ + H_2_O

110 6H_2_O + 6Fe^2+^ + O_2_ → 12H^+^ + 2Fe_3_O_4_

After the reaction, iron oxides and hydroxides Fe_3_O_4_, Fe(OH)_3_, and Febo(OH) were produced. Furthermore, a substantial drop in the peak extent of Fe^0^ was observed, suggesting partial Fe^0^ reaction with NB. A potential pathway for oxygen transport was suggested here: Initially, the generated water molecules received oxygen from the nitro group, eqn (109)–(112).

111 ArNO_2_ + 3Fe^0^ + 6H^+^ → ArNH_2_ + 3Fe^2+^ + 2H_2_O

112 ArNO_2_ + 6Fe^2+^ + 6H^+^ → ArNH_2_ + 6Fe^3+^ + 2H_2_O

Thereafter, the molecules of water that had generated at the composite border interacted predominantly with Fe^2+^ and Fe^3+^ and transferred oxygen to FeO, eqn (113)–(115), which were subsequently coated on the BC interface to prevent the sites of action from being exposed. Oxygen was transferred to the SO_4_^2−^ instantaneously by the reaction of iron sulfides (FeS_*x*_) with water, eqn (116) and (117). Moreover, Fe^3+^ might oxidize FeS_2_ indirectly and by eqn (91) with greater efficiency compared to O_2_, and the resulting Fe^2+^ decreased NB to AN in accordance with eqn (118). Therefore, FeS_2_ increased Fe^3+^/Fe^2+^ cycle and NB reduction by S-nZVI@BC_3 : 1_.^205^

113 Fe^3+^ + 3OH^−^ → FeO(OH) + H_2_O

114 4Fe^0^ + 8OH^−^ + O_2_ + 2H_2_O → 4Fe(OH)_3_

115 2FeS_2_ + 2H_2_O + 7O_2_ → 2Fe^2+^ + 4SO_4_^2−^ + 4H^+^

116 FeS_2_ + 14Fe^3+^ + 8H_2_O → 15Fe^2+^ +2SO_4_^2−^ + 16H^+^

117 2FeS + O_2_ + 2H_2_O → 2Fe(OH)_2_+2S^0^

118 4S^0^ + 2H_2_O → 3S^2−^ + SO_4_^2−^ + 8H^+^

##### 2.1.9.6. Effect of [S-nZVI@BC] dosage on NB removal efficiency

By increasing the composite dose from 2.5 to 10 mg g^−1^, the NB elimination rate enhanced significantly to >98% and stabilized when the amount being administered was raised again to 15 mg g^−1^. In order to remove NB as effectively as possible, S-nZVI@BC_3 : 1_ of 10 mg g^−1^ was chosen. When the composite dose was increased from 2.5 mg g^−1^ (*K*_obs_ = 0.1949 h^−1^) to 10 mg g^−1^ (*K*_obs_ = 6.6062 h^−1^), the NB was promptly eliminated. The primary explanation could be that increasing the amount of Sulfonated zero valent iron modified biochar (S-nZVI@BC_3 : 1_) increased the particular surface area available for additional chemical interaction locations, which in turn increased the effectiveness of interaction frequencies among NB & composites Fig. 8(E).^206,207^ The electron supplies from Sulfidated zero valent iron modified biochar (S-nZVI@BC_3 : 1_) of 5 mg g^−1^ was hypothetically approximately twice that of the balanced demand, as stated in eqn (84). Hence, fast sorption of Sulfonated zero valent iron modified biochar (S-nZVI@BC_3 : 1_) on soil may be the cause of poor NB elimination efficiency since it can decrease Sulfidated zero valent iron modified biochar (S-nZVI@BC_3 : 1_) movement and lessen the likelihood of Sulfidated zero valent iron modified biochar (S-nZVI@BC_3 : 1_) and pollutant contact. Nevertheless, regardless of the Sulfidated zero valent iron modified biochar (S-nZVI@BC_3 : 1_) dosage, the outcome achieved equilibrium in 6 hours.

## 3. Conclusion

This comprehensive review highlights the significant advancements in the use of nano zero-valent metals (nZVMs) for the degradation of pesticides through advanced oxidation processes (AOPs), with particular emphasis on persulfate activation. The integration of nZVMs such as nano zero-valent iron (nZVI), zinc (nZVZn), and copper (nZVCu) into AOPs has proven to be highly effective in accelerating the degradation of persistent organic pollutants, including various pesticides. The elucidation of underlying mechanisms, such as electron transfer and the generation of reactive oxygen species (ROS), has provided crucial insights into the processes driving these degradation reactions. Operational parameters, including pH, catalyst dosage, and reaction time, were found to significantly influence the efficiency of pesticide removal, and synergistic approaches that combine nZVMs with other catalytic systems have further enhanced their degradation capabilities. Additionally, composite systems, such as nZVI/BC and nZVZn/PMS, have shown promising improvements in catalyst stability, dispersibility, and overall degradation efficiency.

Despite the promising results, challenges such as catalyst recovery, the environmental impact of by-products, and the scalability of these systems for industrial applications remain. Addressing these issues will be essential to ensure the successful application of nZVMs in large-scale environmental remediation efforts. Future research should focus on refining the synthesis of novel nZVM composites, developing standardized testing protocols, and evaluating the long-term environmental implications of pesticide degradation products. Overall, this review underscores the importance of eco-friendly, sustainable technologies in addressing the growing environmental concerns posed by pesticide contamination, with nZVM-based AOPs representing a promising path forward for effective and efficient pesticide remediation.

## Conflicts of interest

There are no conflicts to declare.

## Supplementary Material

RA-015-D5RA06043E-s001

## Data Availability

The data supporting the findings of this study are available from the corresponding author upon reasonable request. Supplementary information is available. See DOI: https://doi.org/10.1039/d5ra06043e.
