# Supplementary material for: Advanced oxidation processes for pesticide degradation: a comprehensive review on the role of nano zero-valent metals and persulfate activation
Source: RSC Adv. 2025 Oct 27;15(48):40619–54. doi: 10.1039/d5ra06043e (PMC12557329; doi:10.1039/d5ra06043e)
Supplement: RA-015-D5RA06043E-s001 [file RA-015-D5RA06043E-s001.pdf]

Supplementary Information (SI) for RSC Advances.
This journal is © The Royal Society of Chemistry 2025

Table Caption

Table S1: Pirimicarb intermediates analysed during the Photo-Fenton-nZVI treatment

Compound	Main peak m/z	Refrences
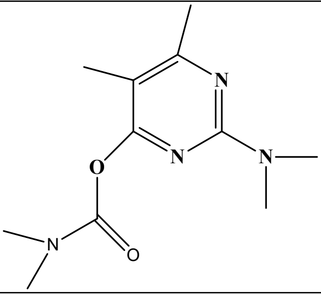	137, 195, 239	[60, 62]
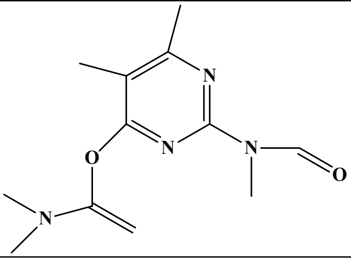	275	[60, 63]
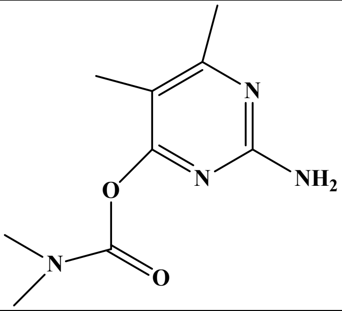	154, 166	[60]
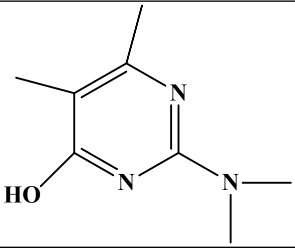	123, 98, 72	[60, 64]
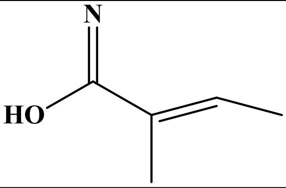	98	[60, 65]

[63].

Table S2; CPY and total chlorine percent removals for each metal tested after 30 days of reaction. $[CPY]_0 = (103.0 \pm 4.5)$ mg L^{-1}; initial metal concentration = 0.5% (w/v); $pH_0 = 6.0 \pm 0.2$ [85].

Metal	CPY Removal (%)	Total chlorine removed (%)
ZVZ/Cu	>99.9	16.9
ZVZ	24.4	6.3
nZVI/Cu	90.0	12.2
nZVI	43.3	5.3
mZVI	19.0	3.8
mZVI/Cu	>99.9	10.0
ZVC	>99.9	21.4

Table S3; Overview of the major studies dealing with atrazine degradation during the last 5 years (2017-2021) along with applied methods [117].

Method	Media	Atrazine dose	Degradation efficiency	Refrences
Persulphate (PS) activated via nanoscale zerovalent iron (nZVI) and Graphene (GR) composite	Water	10 mg L^{-1}	92.1% within 21 minutes	[69]
Cobalt-mediated activation of peroxymonosulphate (PMS)	Water	10 µM	0.225 per minute	[74]
Magnetic Fe_3O_4-seplolite composite	Water	10 mmol L^{-1}	71.6 in 60	[75]

			minutes	
Aerobic zero-valent aluminium (ZVAl/Air) and zero-valent iron (ZVI/Air)	Water	20 mg L^{-1}	Maximum 96.3	[76]
Activation of peroxymonosulphate via cobalt impregnated biochar	Water	10 µM	From 0.76 to 0.36 /minute	[77]
Activation of peroxymonosulphate via $CoNi_3O_4$/diatomite Hybrid	Water	5-ppm	>93% within 30 minutes	[78]
Persulphate coupled with dithionate	Water	1.0 µM	100% within 90 minutes	[79]
Persulphate activated by Pyrite	Water	20 mg L^{-1}	70% within 10 minutes	[80]
Photocatalytic degradation of Atrazine using a UV reactor and UV/MW electrodeless discharge lamp (Hg-EDL)	Water	3.0 mg L^{-1}	3.35 mg L^{-1} in 20 minutes	[81]
Co/Sm Co-modified Ti/PbO_2 anode	Water	20 mg L^{-1}	92.6 within 3 hr	[82]
Iron catalyzed photo-activation of persulphate ($UV/PS/Fe^{2+}$) under mercury-free KrCl	Water	4.0 mg L^{-1}	90% within 15 minutes	[83]

irradiation (222 nm)				
Fe_3O_4/ PMS system in the presence of Hydroxalamine (HA)	Water	23 µM	94% within 15 minutes	[84]

Method	References
Cu-ZnO integrated with g-C_3N_4 to create Cu-ZnO/ g-C_3N_4 Z-direct scheme photocatalyst for advance atrazine removal	[85]
Ferrate (Fe(IVI)) peroxymonosulphate (PMS) processes	[69]
Nano sized $Ba_{1-x}Cu_xO_3$ powder	[86]
CoMgAl layered double oxide catalyzed peroxymonosulphate	[87]
Bioaugmentation with *pseudomonas* and *Arthrobacter*	[88]
$ZnIn_2S_4$-based catalysts	[89]
Biodegradation of Atrazine by the novel *Citricoccus sp*. Strain by TT3	[90]
Bioremediation with Arthrobacter sp. ZXY-2	[91]
CdS/BiOBr/$Bi_2O_2CO_3$ ternary hetero-structure materials	[92]
Bentoine-supported nZVI (β-nZVI) as a catalyst to activate H_2O_2 in the presence of FeS_2	[93]
Zero-valent iron and biochar composite (ZVI/BC) activated persulphate (PS)	[94]
Enhanced electro-Fenton performed by fluorine porous carbon	[95]
Dielectric Barrier Discharge (DBD) method	[96]

Table S4; Degradation of Various pesticides through Nanoscale zero valent iron [134].

Catalyst	Pesticide	Reaction condition	Degradation efficiency (%)	Ref
1;ZVI/(Fe^0) commercial product	Chlorpyrifos 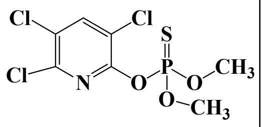	5mgL^{-1} with 1.0% (w/v) ^{ZVI} By adding 1.0% (w/v) $FeCl_3$ Persulphate–ZVI system	43.6<br>71.2<br>100	[91]
2; NZVIs ($nZVI_{20}$), $nZVI_{50}$, $nZVI_{100}$	Pentachlorophenol 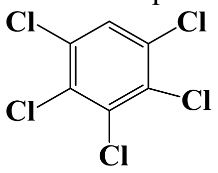	PCP contaminated soil with 100mg nZVI/kg soil	83.9-89.0	[93]
3; nZVI	DDT 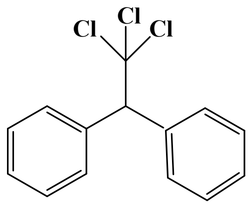	5% w/w nZVI was added to the soil spiked with DDT	96.95	[94]
4; nZVI @CS-PS	Atrazine 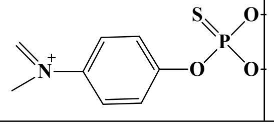	nZVI@CS-800 dose increased from 50 mg L^{-1} to 100 mg L^{-1}	73.92-84.27	[94]

Catalyst	Pesticide	Degradation condition	Degradation efficiency (%)	Ref
5; Zero-valent iron (ZVI)	Methyl parathion 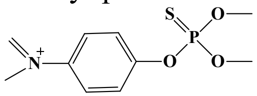	particle size = 0.8-0.3mm, surface area = 0.5 $m^2 g^{-1}$, ZVI loading was varied 50, 200, 400 g L^{-1}	40-100	[95]
6; zero valent iron	Imidacloprid 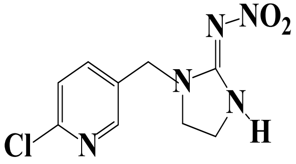	Light source; solar, $S_2O_8^{-1}$=1Mm, ZVI= 55.8-500 mg, Reaction time <60 min	>80	[96]
7; Nano scale zero valent iron	2,4-dichlorophenoxyacetic acid. (2,4-D) 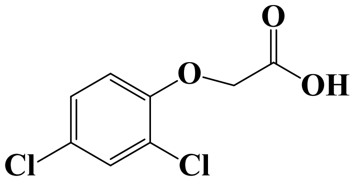	2,4-D concentration=0.1mM, nZVI=0.2g L^{-1} or 1g, Treatment time=120 Min	65-93	[97]

8; Zero valent iron	Dichlorodiphenyltrichloroethane(DDT) 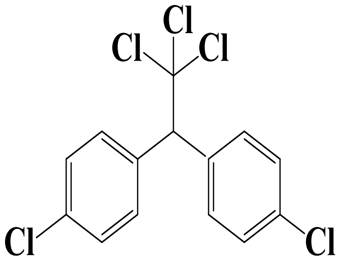	Labortory media =contaminated soil; mean conc of DDT and dieldrin in the soil were 13000±6900 ng and 400±290 ng respectively	75-80	[98]
9; Bio zero valent iron (ZVI)	Chlorpyrifos 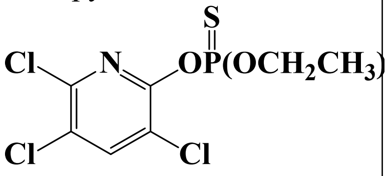	Aerobic bioreactor shaving reactors=1 gL^{-1}; initial CP conc=800µg L^{-1}.	94.5	[99]
10; Zero valent iron (ZVI)	Imidacloprid 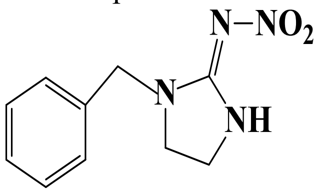	Imidacloprid wastewater=100 ml; at initial P^{H}=3; catalyst=5 g L^{-1}; reaction time=4 h	96.8	[89]

Table S5; Summary of physical and chemical methods for the synthesis of NZVI [139].

Methods	Raw materials	Advantages	Shortage	References
**Precision milling**	Micro iron powder	No Toxic	Energy consumption	[109]
**Lithography**	Bulk iron Material	Inexpensive	Limited control over particle size	[110]
**Ball Milling**	Micro scale FeO	Control size & specific area	Defectiveness	[111]
**EWE**	Wire of metals and metals Alloys	Simple, High surface	Weak aggregation,	[112]
**Liquid Reduction**	$NaBH_4$+Fe^{2+}/Fe^{3+} + salt solution	Homogeneity; High reactivity	High cost; toxic by-products	[113]
**Carbothermal Reduction**	$Fe(CO)_5$	Cheap; Easily available	High Energy, strict conditions	[100]
**Ultrasound assisted Reduction**	$NaBH_4$+Fe^{2+}/Fe^{3+} + salt solution	Uniform; small particle size	Toxic by-products	[114]
**Electrochemical**	Fe^{2+}/Fe^{3+} + salt solution + electrodes	Simple; Cheap; Fast	Strong tendency of aggregation	[115]
**Gas phase thermal reduction**	H_2+goethite/hematite particles	Less By-products	Strict conditions; High temperature	[108]
**Reactive precipitation**	$FeCl_2.4H_2O$ + $NaBH_4$	Convenient; Low Cost	Byproducts; Wastewater; Toxic	[116]
**Green synthesis**	Plant extracts + Fe^{2+}solution	Clean; Eco-friendly Cheap	Poor production	[117]

STRUCTURE	MOLECULAR FORMULA	HPLC R_t	m/z SIGNAL	METAL SYSTEM
**F; (2E)-4-amino-3-chloro-4-oxo-2-enoic acid** STRUCTURE	$C_9H_{11}NO_3PSCl_3$ MOLECULAR FORMULA	9.5 HPLC R_t	305.99 m/z SIGNAL	ZVC METAL SYSTEM
**A; O, O-diethyl-o-(3,5,6-trichloropyridin-2-2-)-phosphothioate** 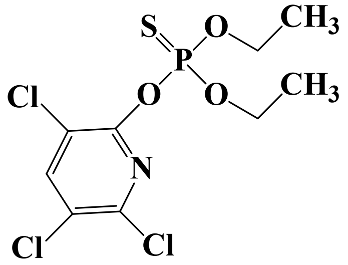	$C_{12}H_{14}Cl_3NO_3PS$	22.8	349.5	
**B; 3,5,6-Trichloropyridinol** 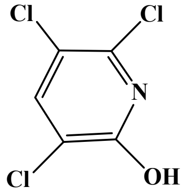	$C_5H_2NOCl_3$	11.1	195.2	nZVI, nZVI/Cu, ZVZ, nZVZ/Cu
**C; Phosphorothioic Acid** 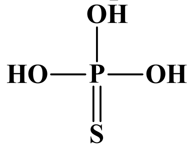	H_3O_3PS	11.85	112.9	nZVI, nZVI/Cu, ZVZ, nZVZ/Cu
**D;O-(4-chloropyridin-2-yl) phosphinothioate** 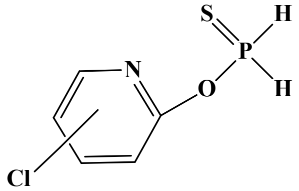	$C_5H_5NOPSCl$	10.6	191.97	ZVC
**E; O-(3-chloropyridin-2-yl O-ethyl O-methyl phosphothioate** 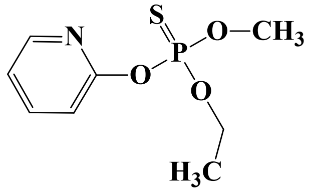	$C_9H_{11}NO_3PSCl_3$	9.5	305.99	ZVC

Table S6: By using LC/ESI(+)-IT-TOF and LC/ESI (-)-IT-TOF analyses on treatedsolutions, chlorpyrifos and its breakdown products identifications [85].

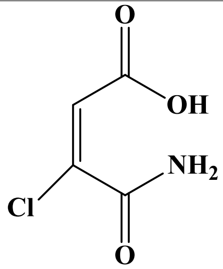				
G; 6-chloropyridin-2-oL 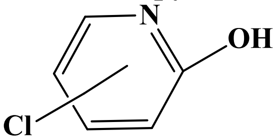	C_5H_4ONCl	7.1	169.08	ZVZ
H; O-pyridin-2-yl dihydrogen phosphorothioate 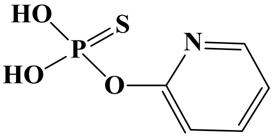	$C_5H_6NO_3$	6.57	191.05	ZVZ/Cu
I;Cyclohexa-2,4-dien-1-ol 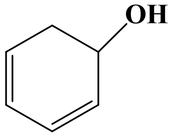	C_6H_8O	6.39	96.97	nZVI
J; Diethylthiophosphate (DEP) 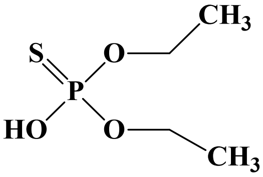	$C_2H_{11}O_3PS$	6.8	169.01	nZVI/Cu

STRUCTURE	MOLECULAR FORMULA	HPLC R_t	m/z SIGNAL	METAL SYSTEM
K;O,O-diethyl-o-(3,5,6-tri chloropyridin-2-yl)-phosphothioate 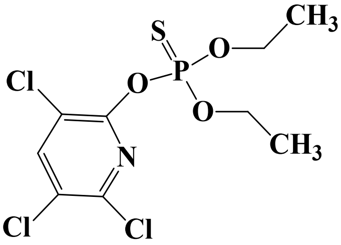	$C_7H_70_3$	7.83	319.89	ZVZ

TABLE S7; Estimated contribution of reaction involved in NB removal [225].

Processes	Reaction	K_{obs}×10^{-2} min^{-1}	R^2	Contribution %
E-Fe^0-O_3	A+B+C+D	11.3	0.99	100
E-Fe^0-O_3(MeOH)	B+C+D	0.84	0.99	_
_	A	_	_	92.8
Electrolysis(MeOH)	B	0.39	0.99	3.3
Ozonation(MeOH)	C	0.23	0.98	1.6
_	D	_	_	2.2
